# Supplementary material for: Myeloid Growth Factors Promote Resistance to Mycobacterial Infection by Curtailing Granuloma Necrosis through Macrophage Replenishment
Source: Cell Host Microbe. 2015 Jul 8;18(1):15–26. doi: 10.1016/j.chom.2015.06.008 (PMC4509513; doi:10.1016/j.chom.2015.06.008)
Supplement: Document S1. Figures S1–S5 [file mmc1.pdf]

Cell Host & Microbe, Volume *18*

Supplemental Information

Myeloid Growth Factors Promote Resistance to Mycobacterial Infection by Curtailing Granuloma Necrosis through Macrophage Replenishment

Antonio J. Pagán, Chao-Tsung Yang, James Cameron, Laura E. Swaim, Felix Ellett, Graham J. Lieschke, and Lalita Ramakrishnan

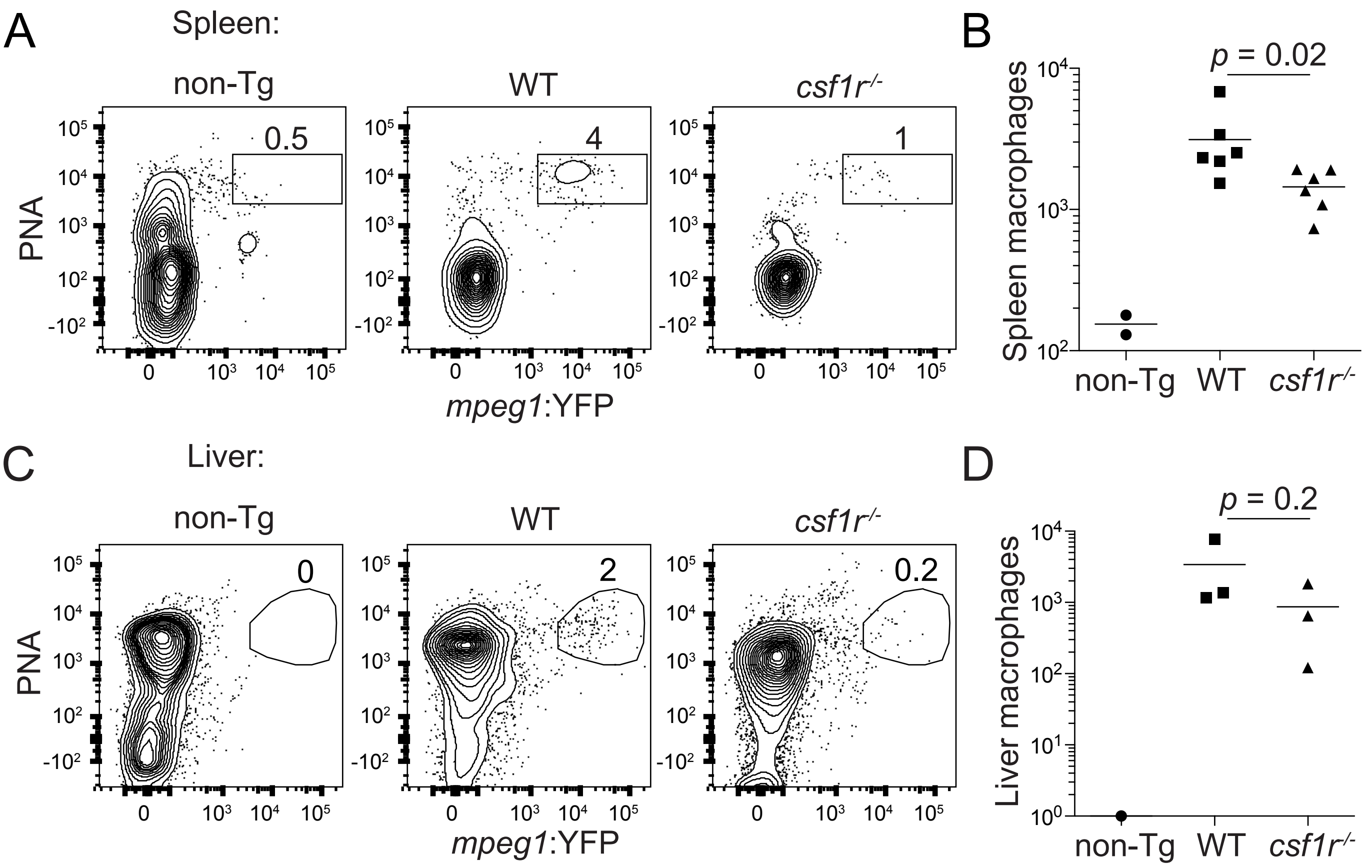

Figure S1. (Related to Figure 1). Adult csf1r mutant zebrafish have impaired macrophage development. (A) Flow cytometry plots and (B) absolute numbers of viable (DAPI$^-$) splenic macrophages in 3-4 month-old wild-type (WT) and *csf1r$^{-/-}$ mpeg1:YFP* fish. *mpeg1:YFP$^-$* WT (non-transgenic, non-Tg) fish were used as negative controls. Each symbol represents individual larvae. (C) Flow cytometry plots and (D) absolute numbers of viable (DAPI$^-$) liver macrophages in 3-4 month-old wild-type (WT) and *csf1r$^{-/-}$ mpeg1:YFP*. Numbers above gated populations indicate percentages within parent population. Data are representative of two independent experiments (A) or were pooled from two experiments (B). Statistical significance determined by two-tailed unpaired Student's t test.

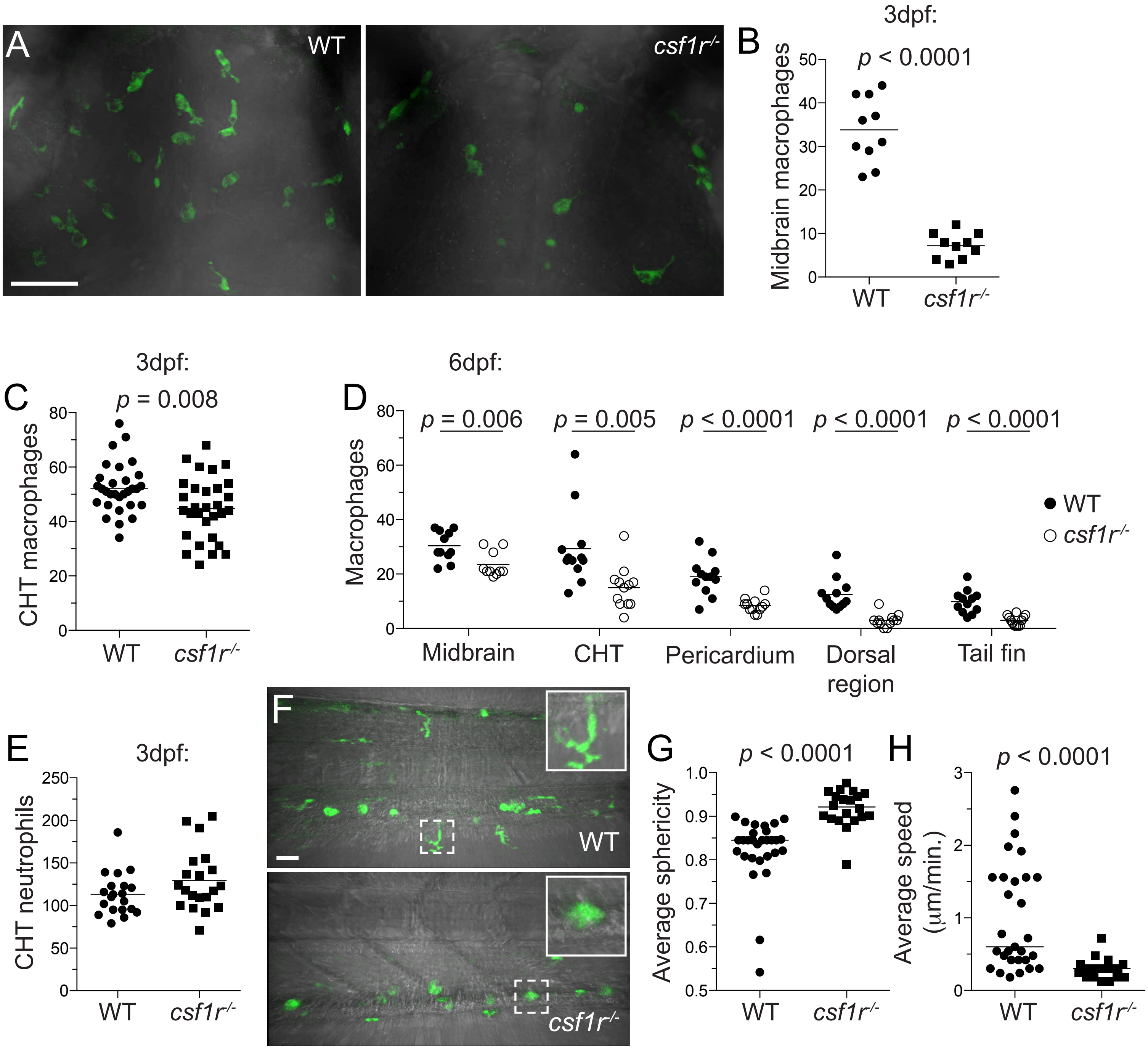

Figure S2. (Related to Figure 1). Larval csf1r mutant zebrafish have impaired macrophage development. Enumeration of larval macrophages in wild-type (WT) and *csf1r-/- mpeg1:YFP* fish. (A) Maximum intensity projections of the hindbrains of WT and *csf1r-/- mpeg1:YFP* larvae at 3dpf. Scale bars, 100μm. Number of macrophages in the (B) midbrain and (C) CHT of 3dpf fish. (D) Absolute numbers of macrophages at 6dpf. Number of neutrophils in the CHT of 3dpf fish as determined by Sudan Black staining. (B - E) Each symbol represents individual larvae. (B - D) Data are representative of at least two experiments. (F - H) Assessment of macrophage motility: (F) Representative maximum intensity projections of the CHT of WT and *csf1r-/- mpeg1:YFP* larvae at 6dpf and (G) average sphericity and (H) speed of individual macrophage tracks. Hashed boxed indicate zoomed region displayed on right corner. Scale bar, 40μm. Horizontal lines indicate means (B - E) or median values (G, H). Statistical significance determined by two-tailed unpaired Student's t test (B - E) or Mann-Whitney U test (G, H).

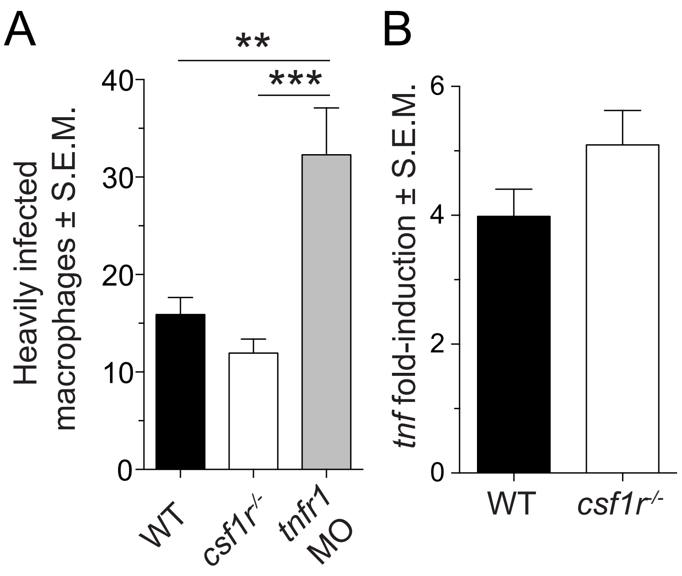

Figure S3. (Related to Figure 1). *csf1r* mutant macrophages have normal microbicidal function during mycobacterial infection. (A) Mean percentage of heavily infected macrophages in WT, *csf1r* mutant, and *tnfr1* morphant larvae 2dpi with ~186 *Δerp M. marinum*. n = 9 or 10 per group. (B) Mean induction of *tnf* expression in WT and *csf1r* mutant larvae 3dpi via the caudal vein with ~220 *M. marinum* over mock-infected, genotype-matched controls. Horizontal lines depict means. Data were pooled from three independent experiments. n = 3 biological replicates per group, each consisting of ~30 larvae. (A) One-way ANOVA with Tukey's post-test or (B) two-tailed unpaired Student's t test with Welch's correction was performed to determine statistical significance.

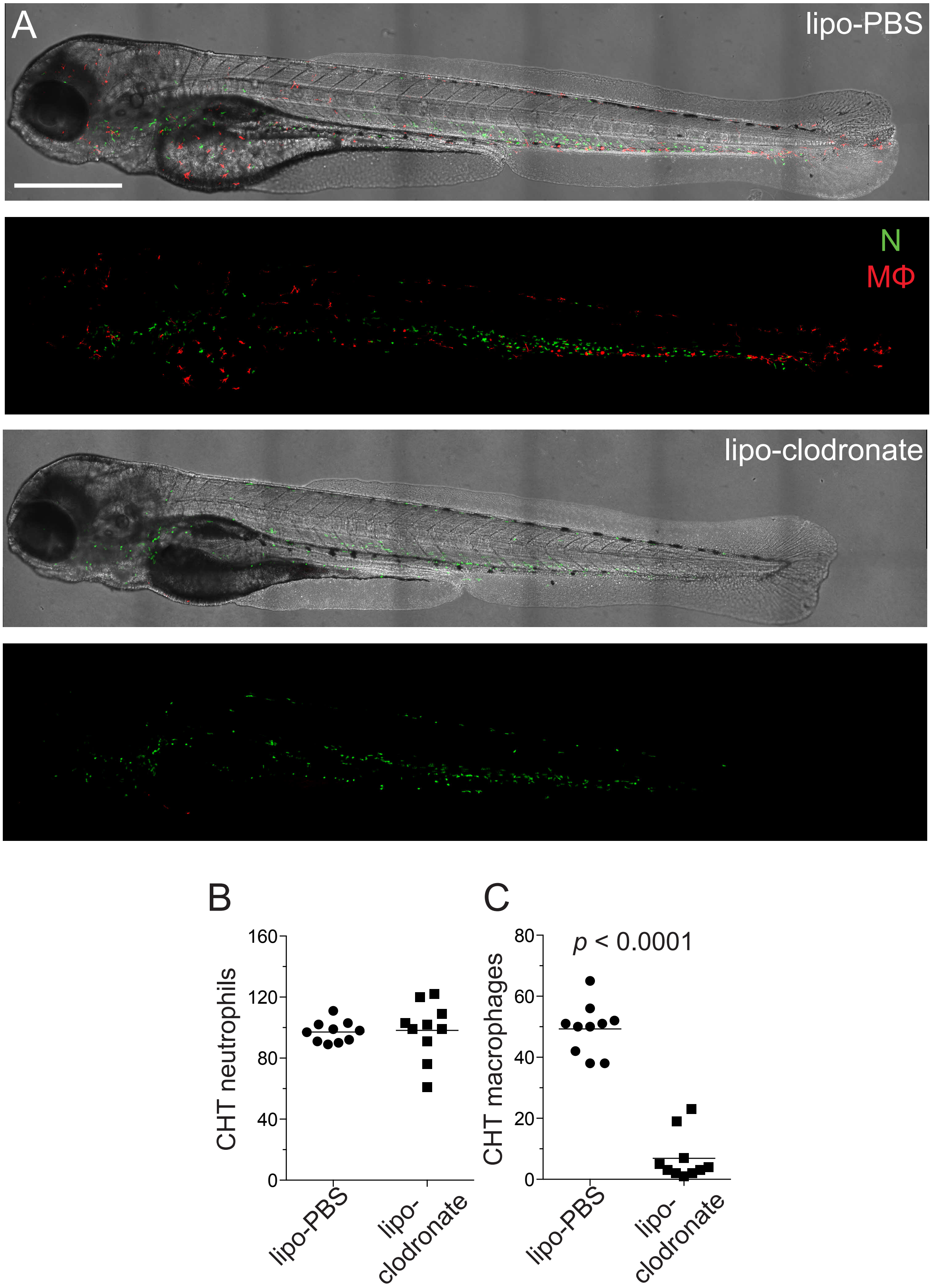

Figure S4. (Related to Figure 3). Lipo-clodronate treatment specifically depletes macrophages in zebrafish. (A) Stitched, 80μm maximum intensity projections of 4dpf *lysC:EGFP*; *mpeg1:tdTomato* larvae 40 hours after intravenous injection with 1:5 dilutions of lipo-PBS (left) or lipo-clodronate (right). $EGFP^+$ neutrophils are shown in green and $tdTomato^+$ macrophages are depicted in red. Scale bar, 500μm. Numbers of neutrophils (B) and macrophages (C) in the CHT of 4dpf *mpeg1:tdTomato* larvae 40 hours after liposome injection.

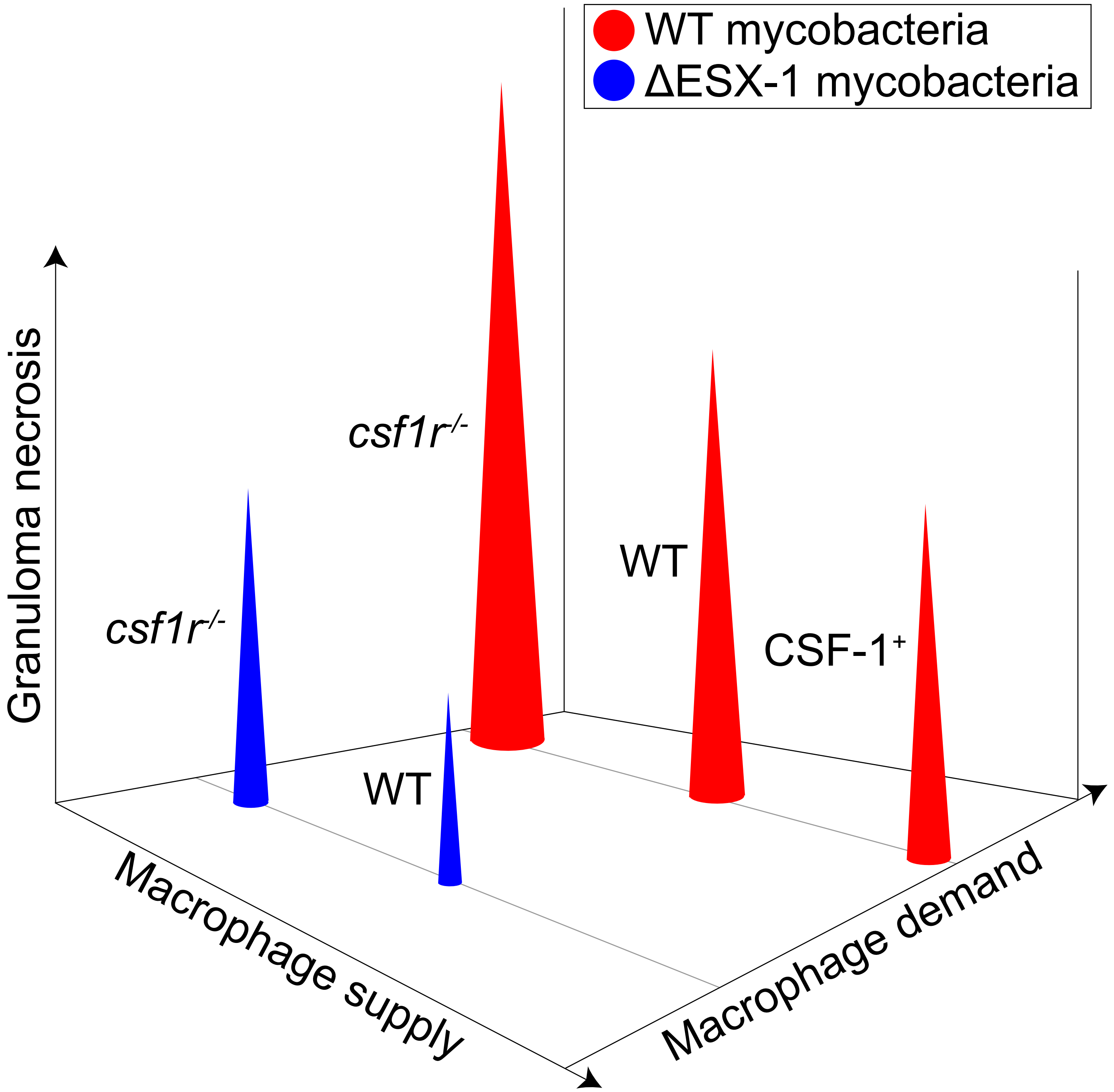

Figure S5. (Related to Figures 2 - 4 and Figure 6). Macrophage demand and supply interact to modulate granuloma necrosis. The relative effects on granuloma necrosis of concurrent variations in macrophage supply and demand tested in this study are shown in a cartoon graph.
