# Supplementary figures and images for: Myeloid Growth Factors Promote Resistance to Mycobacterial Infection by Curtailing Granuloma Necrosis through Macrophage Replenishment
Source: Cell Host Microbe. 2015 Jul 8;18(1):15–26. doi: 10.1016/j.chom.2015.06.008 (PMC4509513; doi:10.1016/j.chom.2015.06.008)

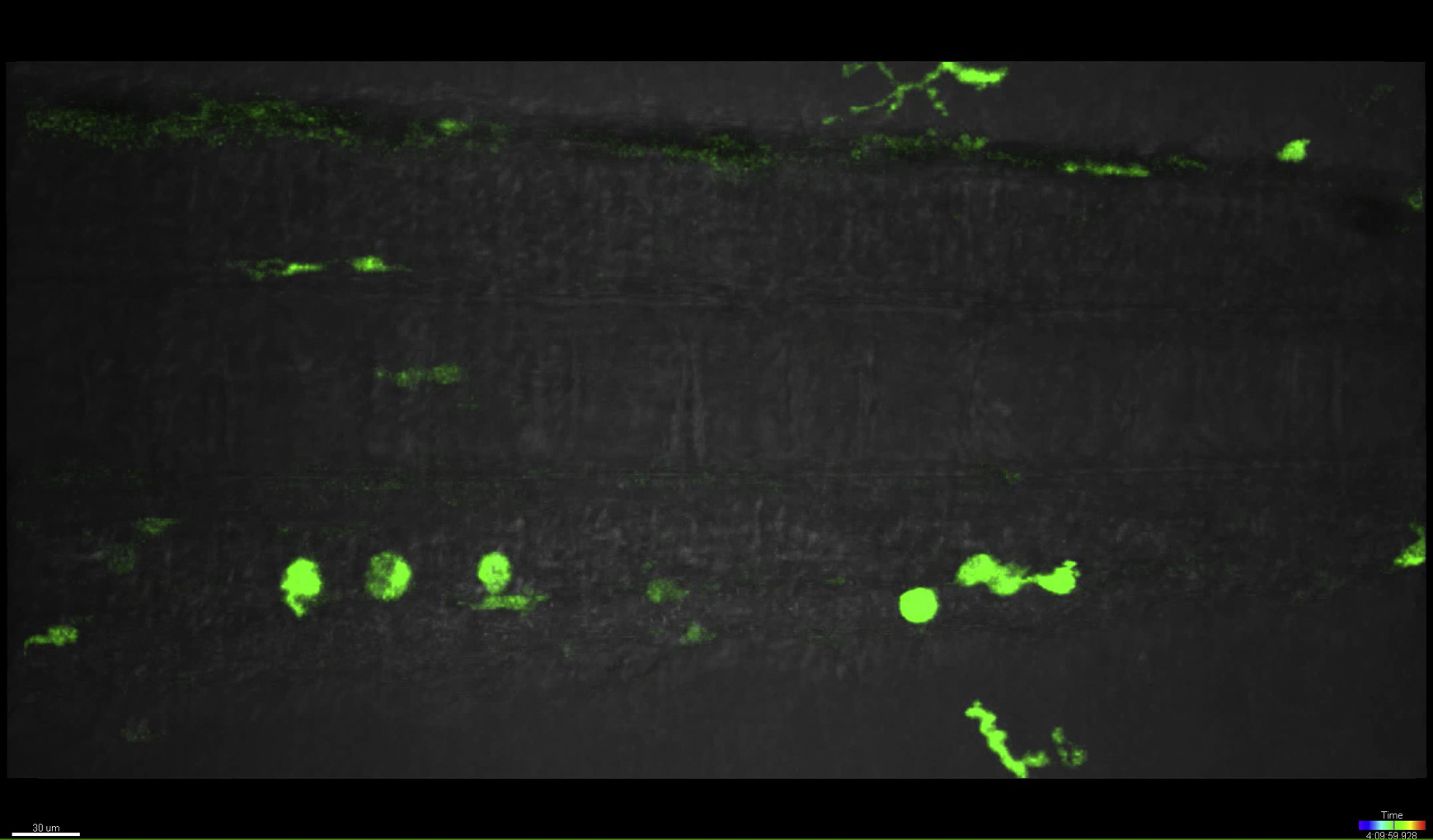

Supplement: Movie S1. Motility of Macrophages in Wild-Type Zebrafish Larva — Video shows macrophages (green) in the CHT of a 6-dpf wild-type mpeg1:YFP fish. Related to Figure 1 and Figure S1. [file mmc2.jpg]

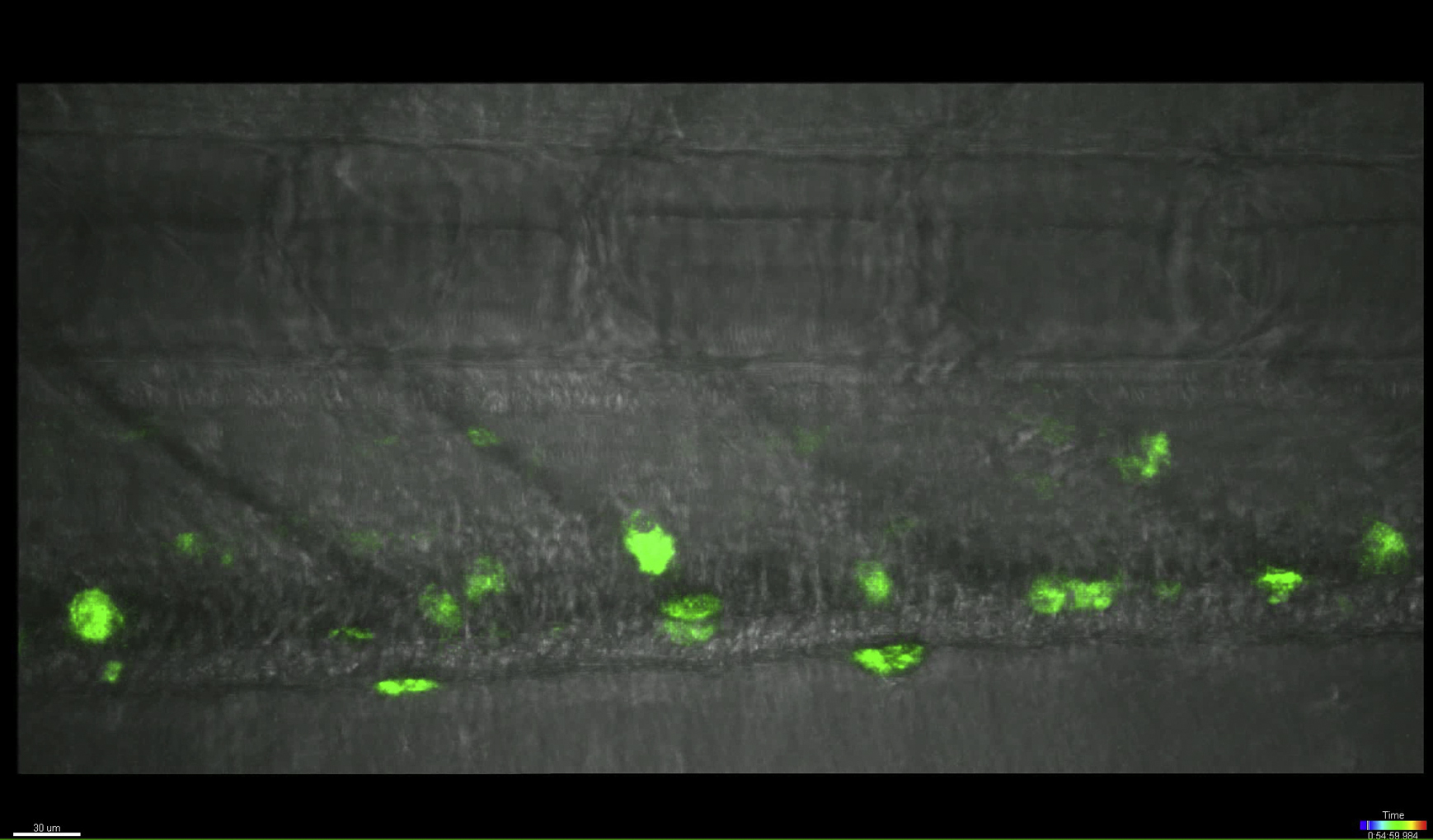

Supplement: Movie S2. Motility of Macrophages in csf1r Mutant Zebrafish Larva — Video shows macrophages (green) in the CHT of a 6-dpf csf1r-/- mpeg1:YFP fish. Related to Figure 1 and Figure S1. [file mmc3.jpg]
