# Supplementary material for: Myeloid Growth Factors Promote Resistance to Mycobacterial Infection by Curtailing Granuloma Necrosis through Macrophage Replenishment
Source: Cell Host Microbe. 2015 Jul 8;18(1):15–26. doi: 10.1016/j.chom.2015.06.008 (PMC4509513; doi:10.1016/j.chom.2015.06.008)
Supplement: Document S2. Article plus Supplemental Information [file mmc4.pdf]

Article

Cell Host & Microbe

Myeloid Growth Factors Promote Resistance to Mycobacterial Infection by Curtailing Granuloma Necrosis through Macrophage Replenishment

Graphical Abstract

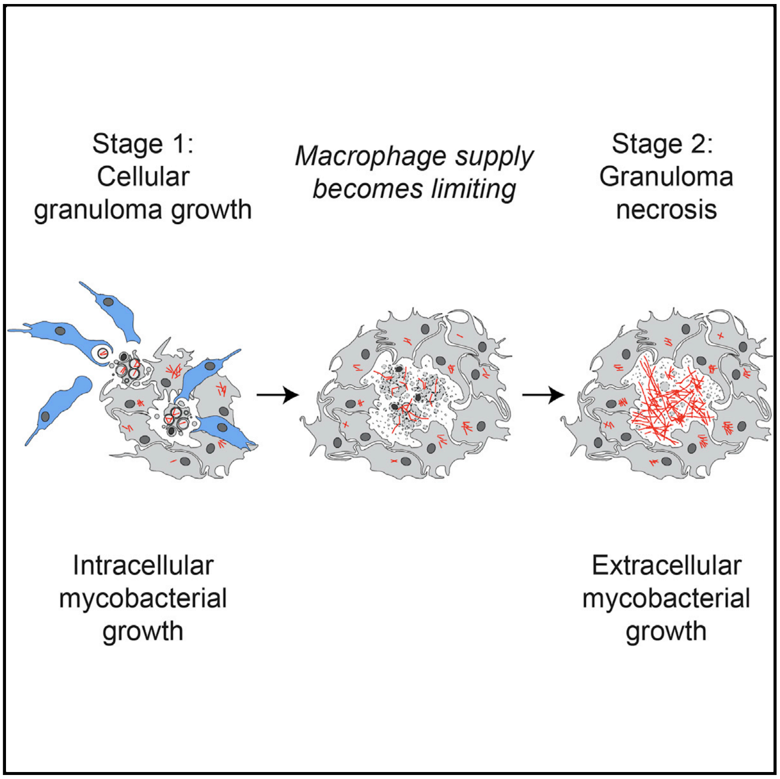

Authors

Antonio J. Pagán, Chao-Tsung Yang,
James Cameron, ..., Felix Ellett,
Graham J. Lieschke,
Lalita Ramakrishnan

Correspondence

lr404@cam.ac.uk

In Brief

The early tuberculous granuloma promotes intracellular bacterial growth by co-opting host macrophages to serve as bacterial growth niches. Pagán et al. show that, conversely, an adequate macrophage supply to the granuloma is required to prevent necrosis of infected macrophages and subsequent extracellular bacterial growth.

Highlights

- Myeloid deficiencies increase innate immune susceptibility to mycobacterial infection
- Depletion of macrophage supply below a critical threshold hastens granuloma necrosis
- Increasing macrophage supply to the granuloma delays granuloma necrosis

Pagán et al., 2015, Cell Host & Microbe *18*, 15–26
July 8, 2015 ©2015 The Authors
http://dx.doi.org/10.1016/j.chom.2015.06.008

Myeloid Growth Factors Promote Resistance to Mycobacterial Infection by Curtailing Granuloma Necrosis through Macrophage Replenishment

Antonio J. Pagán,[1,2,7] Chao-Tsung Yang,[2,7,8] James Cameron,[2,9] Laura E. Swaim,[2,10] Felix Ellett,[3] Graham J. Lieschke,[3,4] and Lalita Ramakrishnan[1,2,5,6,*]

[1]Department of Medicine, University of Cambridge, Cambridge CB2 0QH, UK
[2]Department of Microbiology, University of Washington, Seattle, WA 98195, USA
[3]Cancer and Haematology Division, Walter and Eliza Hall Institute of Medical Research, Parkville, VIC 3052, Australia
[4]Australian Regenerative Medicine Institute, Monash University, Clayton, VIC 3800, Australia
[5]Department of Immunology, University of Washington, Seattle, WA 98195, USA
[6]Department of Medicine, University of Washington, Seattle, WA 98195, USA
[7]Co-first author
[8]Present address: Janelia Research Campus, Howard Hughes Medical Institute, Ashburn, VA 20147, USA
[9]Present address: Environmental and Fisheries Science Division, National Oceanic and Atmospheric Administration, Seattle, WA 98115, USA
[10]Present address: Skokomish Tribe Department of Fisheries, Shelton, WA 98584, USA
*Correspondence: lr404@cam.ac.uk

http://dx.doi.org/10.1016/j.chom.2015.06.008

This is an open access article under the CC BY license (http://creativecommons.org/licenses/by/4.0/).

SUMMARY

The mycobacterial ESX-1 virulence locus accelerates macrophage recruitment to the forming tuberculous granuloma. Newly recruited macrophages phagocytose previously infected apoptotic macrophages to become new bacterial growth niches. Granuloma macrophages can then necrose, releasing mycobacteria into the extracellular milieu, which potentiates their growth even further. Using zebrafish with genetic or pharmacologically induced macrophage deficiencies, we find that global macrophage deficits increase susceptibility to mycobacterial infection by accelerating granuloma necrosis. This is because reduction in the macrophage supply below a critical threshold decreases granuloma macrophage replenishment to the point where apoptotic infected macrophages, failing to get engulfed, necrose. Reducing macrophage demand by removing bacterial ESX-1 offsets the susceptibility of macrophage deficits. Conversely, increasing macrophage supply in wild-type fish by overexpressing myeloid growth factors induces resistance by curtailing necrosis. These findings may explain the susceptibility of humans with mononuclear cytopenias to mycobacterial infections and highlight the therapeutic potential of myeloid growth factors in tuberculosis.

INTRODUCTION

The pathological hallmark of tuberculosis (TB) is the granuloma, a cellular aggregate consisting of macrophages and other immune cells (Ramakrishnan, 2012). Studies in the *Mycobacterium marinum*-zebrafish model of TB find that despite the ability of participating macrophages to partially restrict mycobacterial growth, the bacteria can actually co-opt the tuberculous granuloma to expand intracellularly (Clay et al., 2008; Davis and Ramakrishnan, 2009; Pagán and Ramakrishnan, 2014). Mycobacteria accomplish this by accelerating recruitment of uninfected macrophages to the growing granuloma. Multiple newly recruited cells phagocytose previously infected macrophages that have undergone apoptotic death.

The mycobacterial ESX-1/RD1 virulence locus promotes both apoptosis of infected macrophages and recruitment of new macrophages to the granuloma (Davis and Ramakrishnan, 2009; Volkman et al., 2004). Accordingly, the attenuated infection by ESX-1 mutant mycobacteria, including the BCG vaccine strain, is characterized by reduced granuloma and bacterial growth (Lewis et al., 2003; Volkman et al., 2004). Thus, the phase of cellular granuloma growth with its attendant intracellular bacterial expansion is driven by a mycobacterium-mediated increase in macrophage demand. It is this increased macrophage demand that converts the granuloma from a protective to a pathogenic entity in this initial cellular phase.

In active human TB, granulomas can progress to central necrosis caused by lysis of the infected macrophages that releases viable mycobacteria into a growth-permissive extracellular milieu (Pagán and Ramakrishnan, 2014). Thus granuloma necrosis can be accompanied by accelerated mycobacterial growth and result in increased disease severity and transmissibility (Cambier et al., 2014). In this work, we have linked granuloma necrosis to a critical reduction in the available macrophage supply to the granuloma. We monitored granuloma formation and fate together with bacterial expansion in zebrafish where macrophage supply was manipulated. We assessed the impact of macrophage deficiency states in animals lacking colony-stimulating factor-1 receptor (Csf-1r) or interferon regulatory factor 8 (Irf8) or after pharmacological macrophage depletion with clodronate-loaded liposomes (lipo-clodronate). Macrophage deficiency states exacerbated granuloma necrosis by accelerating depletion of the macrophage supply. This necrosis could

be delayed by reducing macrophage demand in the granuloma. Conversely, a macrophage surplus created by overexpressing *csf1* in wild-type animals curtailed granuloma necrosis. This work highlights the role of an adequate macrophage supply in increasing host resistance by limiting infected macrophage necrosis and extracellular bacterial expansion.

RESULTS

Zebrafish *csf1r* Mutants Have a Persistent Global Macrophage Deficit from Early in Development

Because mice deficient in CSF-1R signaling have a global macrophage deficit (Cecchini et al., 1994; Dai et al., 2002; Stanley and Chitu, 2014) and are susceptible to *M. tuberculosis* (Teitelbaum et al., 1999), we sought to use CSF-1R-deficient zebrafish to determine the role of macrophage deficiency in TB pathogenesis. To confirm that Csf-1r-deficient zebrafish had fewer macrophages, we generated *csf1ra*^{j4blue} homozygous mutant (*csf1r*$^{-/-}$) zebrafish (Parichy et al., 2000) expressing the macrophage reporter *mpeg1:YFP* (Ellett et al., 2011; Roca and Ramakrishnan, 2013). Similar to CSF-1R signaling-deficient mice, *csf1r* mutant adult zebrafish had fewer spleen and liver macrophages than their phenotypically wild-type *csf1r*$^{+/-}$ siblings (Figure S1) (Cecchini et al., 1994; Dai et al., 2002).

The zebrafish has great utility as a TB model during its larval transparent phase. We therefore asked if Csf-1r deficiency decreases macrophages from early in myeloid development, during the primitive and transient definitive waves of hematopoiesis. Primitive macrophages are specified in the rostral lateral plate mesoderm from 12 hr post-fertilization and then disperse throughout the embryo (Herbomel et al., 1999; Lieschke et al., 2002). A subset of these macrophages migrates to the brain in a Csf-1r-dependent manner to become microglia, the tissue-resident macrophages of the brain (Clements and Traver, 2013; Herbomel et al., 2001). *csf1r* mutant larvae had 80% fewer microglia by 3 days post-fertilization (dpf) (Figures S2A and S2B) (Herbomel et al., 2001), similar to the deficit in mice (Ginhoux et al., 2010).

The second wave of zebrafish hematopoiesis initiates in the caudal hematopoietic tissue (CHT) by 32 hpf before it transitions into the adult kidney through developmental pathways analogous to the transition of hematopoiesis from mouse fetal liver to adult bone marrow (Clements and Traver, 2013; Ginhoux and Jung, 2014). At 3 dpf, *csf1r* mutants had 14% fewer macrophages in the CHT, a deficit that became more pronounced by 6 dpf (49% reduction) and extended to other tissues normally populated by these macrophages (56%, 76%, and 69% reduction in the pericardium, dorsal region, and tail fin, respectively) (Figures S2C and S2D). In contrast, the microglial deficit was less marked at 6 dpf than at 3 dpf, consistent with prior findings (Figure S2D) (Herbomel et al., 2001). Neutrophils were not reduced in *csf1r* mutants (Figure S2E).

Finally, as in mammals, zebrafish *csf1r* mutant macrophages were more spherical and moved more slowly than wild-type macrophages (Figures S2F–S2H and Movies S1 and S2) (Boocock et al., 1989; Sampaio et al., 2011; Stanley and Chitu, 2014; Webb et al., 1996). In sum, zebrafish *csf1r* deficiency recapitulates key features of mammalian CSF-1R signaling deficiency with a persistent deficit in monocyte/macrophage lineage cells as well as phenotypic differences—all from the earliest developmental stages.

Adult and Larval Zebrafish *csf1r* Mutants Are Hypersusceptible to *M. marinum*

We confirmed that adult zebrafish *csf1r* mutants were hypersusceptible to *M. marinum*. Similar to mice lacking CSF-1, a ligand for CSF-1R, they succumbed to infection earlier than wild-type siblings (Figure 1A) (Teitelbaum et al., 1999). Their accelerated mortality was accompanied by higher bacterial burdens (Figure 1B). Because Teitelbaum et al. found fewer mycobacteria transported to the lung-draining lymph nodes in mutant mice, they have argued that CSF-1 deficiency causes susceptibility through impaired *M. tuberculosis*-specific T cell priming (Teitelbaum et al., 1999). If this were the sole defect, then susceptibility should be manifested only in the context of an adaptive immune response. We were able to test this directly in zebrafish larvae, which lack functional lymphocytes (Langenau and Zon, 2005). *csf1r* mutant larvae also had accelerated mortality with increased bacterial burdens (Figures 1C–1E). This finding implicated their macrophage deficit—functional, numerical, or both—as the root cause of hypersusceptibility. To understand this further, we assessed macrophage microbicidal capacity and migration that are both central to early pathogenesis and immunity (Ramakrishnan, 2014).

Macrophages of *csf1r* Mutant Zebrafish Exhibit Normal Microbicidal Capacity In Vivo

We assessed macrophage microbicidal capacity in Csf-1r-deficient versus wild-type animals by enumerating bacterial burdens in individual macrophages 2 days post-infection (2 dpi). Mutant macrophages had similar bacterial burdens to wild-type at this time point (assessment of later time points is precluded by technical limitations) (Clay et al., 2008) (Figure S3A). In contrast, consistent with their known macrophage microbicidal deficit, Tnf receptor1-deficient larvae created by modified antisense oligonucleotide (morpholino) knockdown had higher bacterial burdens in their macrophages (Figure S3A). *csf1r* mutants also had similar *tnf* levels to wild-type at baseline and after infection, consistent with their normal microbicidal capacity (Clay et al., 2008) (Figure S3B and data not shown). Thus, a macrophage microbicidal deficit did not appear to be the underlying mechanism of *csf1r* mutant hypersusceptibility.

csf1r Mutant Granulomas Undergo Normal Cellular Expansion Followed by Accelerated Necrosis

We next assessed the kinetics of granuloma formation in the mutants. Our earlier qualitative observations had suggested that granulomas form normally in *csf1r* mutant fish (Davis et al., 2002), but more quantitative analysis was warranted given our findings that *csf1r*-deficient macrophages moved more slowly under homeostatic conditions. We confirmed that mutant granuloma formation was similar to wild-type up to 4 dpi, showing that the macrophage recruitment in this first phase of granuloma progression was undiminished (Figure 2A and data not shown). This suggests that Csf-1r contributes to homeostatic macrophage migration but not their migration to the tuberculous granuloma. However, thereafter, we noticed a rapid progressive loss of granuloma cellularity accompanied by the characteristic corded

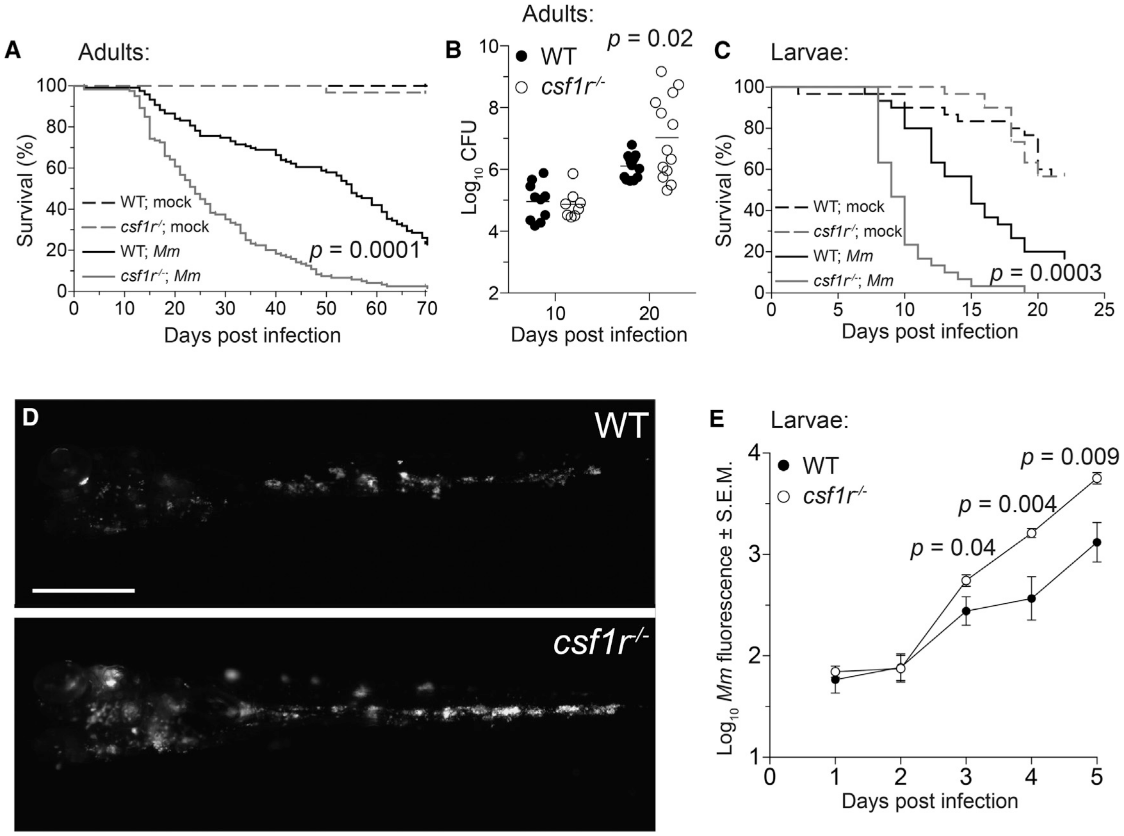

Figure 1. *csf1r* Mutant Zebrafish Are Hypersusceptible to *M. marinum* Infection

(A) Survival of adult zebrafish injected intraperitoneally with ~273 *M. marinum* or an equivalent volume of PBS (mock). n = 30 for each group.

(B) Bacterial burdens (CFU, colony-forming units) of adult zebrafish infected intraperitoneally with ~115 CFU of *M. marinum*. Horizontal lines indicate mean values.

(C) Survival of zebrafish larvae injected with PBS (mock) or ~193 *M. marinum* via the caudal vein. n = 30 for each group.

(D and E) Representative images (D) and mean bacterial burden (E) of larvae infected with ~200 fluorescent *M. marinum* via the caudal vein. Scale bar, 300 μm. Error bars indicate standard error of the mean (SEM).

Statistical significance was determined by log-rank test (A, C) or two-tailed unpaired Student's t test (B, E). Data are representative of more than three experiments. See also Figures S1–S3 and Movies S1 and S2.

bacterial morphology exhibited during extracellular growth in vivo (Figures 2B and 2C) (Clay et al., 2008). Prior work has linked these two phenotypes to the necrosis of infected macrophages where the bacteria are released to grow extracellularly (Clay et al., 2008; Tobin et al., 2010). Taken together, our findings suggested that the first cellular phase of bacterial expansion in the granuloma proceeds normally in the mutant, but the second phase of granuloma necrosis and extracellular bacterial growth occurs earlier. However, it was possible that this conclusion made in larvae, which only have a few hundred macrophages, might not be relevant during a more chronic infection in adult animals with a much more abundant macrophage supply. So we examined granuloma formation and progression in response to *M. marinum* in adult *csf1r* mutant and wild-type siblings. By 2 weeks post-infection, granuloma formation throughout the body was occurring in both groups—macrophage aggregates as well as organized granulomas were seen, but with little evidence of necrosis in either, in accordance with prior studies (Figures 2D and 2E) (Swaim et al., 2006). By 4 weeks, granulomas were more abundant and more uniformly organized in both groups with a similar wide tissue distribution again, as seen previously (Swaim et al., 2006). Wild-type granulomas were still mostly non-necrotic with only a few displaying only partially necrotic areas (Figures 2F and 2G). In contrast, the majority of the Csf1r-deficient granulomas, whether solitary or multicentric, had completely necrotic areas with abundant bacteria (Figures 2H and 2I). In the *csf1r* mutants, we occasionally observed patches of bacteria that were apparently outside of granulomas (e.g., Figures 2H and 2I), a distribution that was absent in the wild-type animals both in this cohort and in prior studies (Figures 2F and 2G) (Swaim et al., 2006). This may be a consequence of bacterial outgrowth from necrotic lesions. These findings corroborate findings in the Csf1r-deficient larvae that granulomas form normally but undergo accelerated necrosis.

Necrosis in *csf1r* Mutants Coincides with a Critical Depletion of the Available Macrophage Supply

We investigated the mechanistic basis of granuloma necrosis in *csf1r* mutants. We have previously shown that TNF dysregulation—both deficiency and excess—causes necrosis of infected

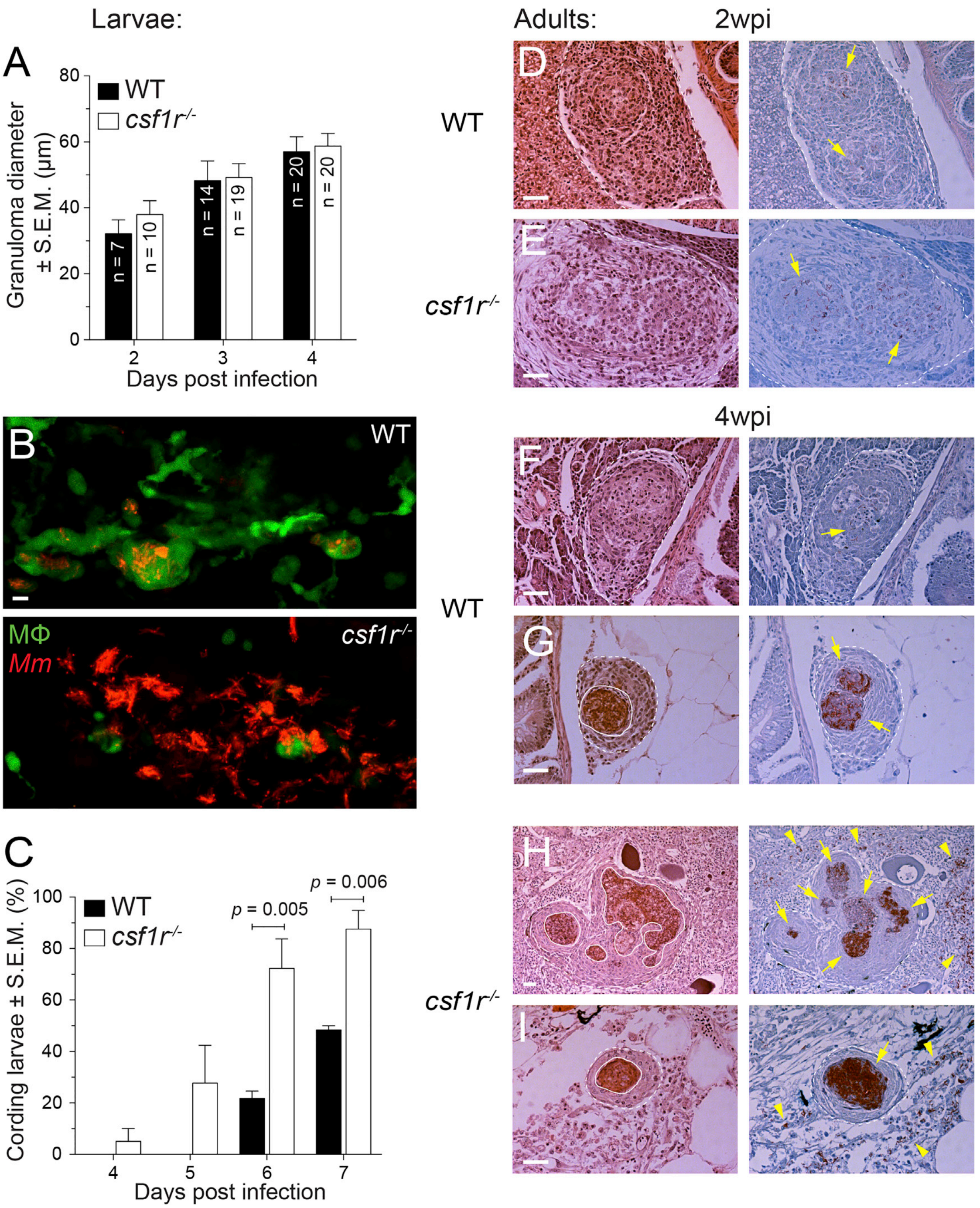

Figure 2. Exuberant Mycobacterial Growth in *csf1r* Mutants Is Associated with Granuloma Necrosis

(A) Mean granuloma diameter measured by their longest axis in larvae infected at 2 dpf via the caudal vein with ~195 tdTomato-expressing *M. marinum*. Data are representative of two sets of injections.

(B) Maximum-intensity projections of macrophage-replete WT granuloma or a macrophage-depleted *csf1r* mutant granuloma showing early signs of cording in 5 dpi *mpeg1:YFP* larvae infected at 2 dpf via the caudal vein with ~200 tdTomato-expressing *M. marinum*. Scale bar, 10 µm.

(C) Percentage of larvae with cording phenotype after infection with ~190 *M. marinum* via the caudal vein. Average values from four separate sets of injections from at least two independent experiments were plotted. In (A) and (C), error bars indicate SEM.

(D–I) Representative hematoxylin and eosin (left) and modified Ziehl-Neelsen (right) stains of wild-type or *csf1r* mutant adult zebrafish 2 weeks (D and E) or 4 weeks (F–I) post-intraperitoneal infection with ~100 CFU of *M. marinum*. Dotted lines delineate granulomas and solid lines indicate regions of necrosis. Arrows indicate mycobacteria inside granulomas and arrowheads indicate mycobacteria outside of granulomas.

Scale bars, 50 µm. Two or three fish per group were used for each time point.

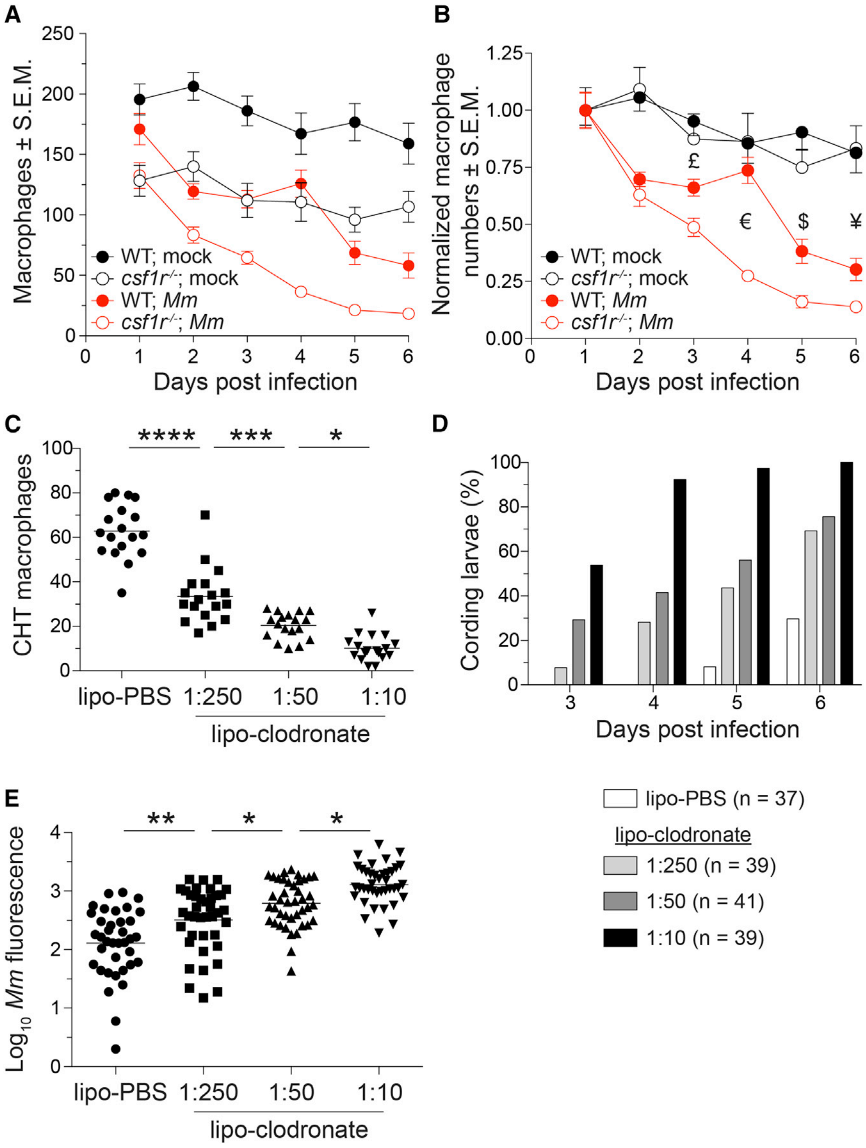

Figure 3. A Baseline Macrophage Deficit Is Associated with an Accelerated Depletion of the Granuloma Macrophage Supply

(A and B) Absolute numbers (A) or normalized numbers (B) of macrophages in *mpeg1:Gal4FF; UAS:E1bKaede* larvae infected with ~240 *M. marinum* or mock injected. n = 5–9 larvae per group. Error bars indicate SEM.

(C) Number of macrophages in the CHT of 4-dpf *mpeg1:YFP* larvae 40 hr after injection of 1:10 dilution of lipo-PBS or graded doses of lipo-clodronate.

(D and E) Bacterial kinetics of cording (D) and burdens (E) in larvae infected with tdTomato-expressing *M. marinum* via the caudal vein at 2 dpf. Statistical significance was determined by two-tailed unpaired Student's t test (B) or one-way ANOVA with Sidak's post-test (C and E). £, $p = 0.02$; €, $p = 3 \times 10^{-8}$; $, $p = 0.004$; and ¥, $p = 0.03$. (C–E) Data are representative of three independent experiments. See also Figure S4.

macrophages (Clay et al., 2008; Roca and Ramakrishnan, 2013; Tobin et al., 2012), but *tnf* induction and microbicidal capacity similar to that of wild-type argues against TNF dysregulation in the *csf1r* mutant as the cause of granuloma macrophage necrosis. Because the phase of intracellular granuloma growth is sustained by a continuous influx of macrophages (Davis and Ramakrishnan, 2009), we wondered if granuloma necrosis could be accelerated by a critical depletion of an already reduced macrophage supply in *csf1r* mutants. If so, we should see a more rapid global macrophage depletion in infected *csf1r* mutants. We enumerated macrophages daily in infected and mock-infected wild-type and mutant animals. As expected, mock-infected *csf1r*-deficient larvae had fewer macrophages at all time points (Figure 3A). Their macrophage deficit remained relatively constant throughout (Figure 3B), consistent with the idea that Csf-1r-derived survival signals are dispensable for maintenance of the macrophage pool, at least under homeostatic conditions during early development. However, following infection, clear differences emerged: in wild-type there was a sharp decline immediately after infection between days 1 and 2 that then slowed down (Figure 3B). This pattern was observed in the mutant also, which had the same initial sharp decline as wild-type (Figure 3B). Although the mutant macrophage decline also slowed thereafter, it remained greater than wild-type in this phase (Figure 3B). These results are consistent with the onset of infection-induced myelopoiesis that is less robust in the mutant than wild-type.

In both mutant and wild-type granulomas, bacterial cording began at time points that coincided with macrophage reduction to below a critical threshold (day 4 and 6, respectively, for mutant and wild-type; compare Figures 2C and 3A). Together, these results suggest that depletion of the available macrophage supply drives necrosis of the granuloma by limiting its macrophage replenishment.

If depletion of the macrophage supply drives granuloma necrosis in the *csf1r* mutants, then we should see a direct correlation between the extent of global macrophage reduction and granuloma breakdown. To test this, we treated wild-type larvae with lipo-clodronate, which depletes macrophages without affecting neutrophils (Bernut et al., 2014) (Figures S4A–S4C). We dose-titrated lipo-clodronate to create graded macrophage reductions: 47% (similar to *csf1r* mutants), 67%, and 84% (Figure 3C). We found that the smaller the macrophage supply, the sooner the development of macrophage necrosis (evidenced by bacterial cording) (Figure 3D). We again confirmed that granuloma necrosis was associated with increased bacterial burdens (Figure 3E). This experiment further implicated a reduction in macrophage supply as the sole driver of granuloma necrosis in the *csf1r* mutants.

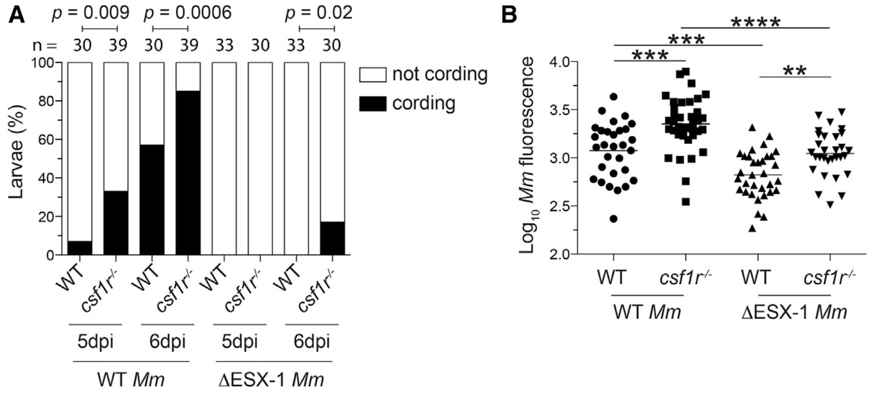

Figure 4. Reducing Macrophage Demand Delays Onset of Granuloma Necrosis

(A and B) 2-dpi larvae were infected via the caudal vein with ~300 WT or ΔESX-1 tdTomato-expressing *M. marinum*. (A) Percentage of wild-type (black bars) or *csf1r*$^{-/-}$ larvae (gray bars) at 5 and 6 dpi. (B) Bacterial burdens at 6 dpi. Each symbol represents individual larvae. Each symbol represents individual larvae, and horizontal lines indicate means. Statistical significance was determined by Fisher's exact test (A) or one-way ANOVA with Sidak's post-test (B). Data are representative of two experiments. See also Figure S4.

Reducing Macrophage Demand Curtails Granuloma Necrosis by Delaying Depletion of the Macrophage Supply

Our results so far suggested that the first cellular phase of granuloma growth occurs when macrophage supply is not limiting and is therefore driven mainly by macrophage demand. However, the quantity of macrophages demanded during the cellular phase should also influence the time of onset of the necrotic phase of the granuloma by influencing the rate of macrophage depletion. To ask if macrophage demand alters the time to granuloma necrosis, we compared wild-type and ESX-1 mutant infection in wild-type animals. ESX-1 mutant granulomas expanded more slowly, as we had shown before (Davis and Ramakrishnan, 2009). By examining infection at later time points, we found that ESX-1 mutant granulomas did not become necrotic, unlike their wild-type counterparts (Figure 4A). Next we tested the effect of reducing both macrophage supply and demand by assessing ESX-1 infection in *csf1r* mutant hosts. In *csf1r* mutants, ESX-1 mutant bacteria produced less necrosis than wild-type bacteria (Figure 4A). However, when we compared ESX-1 mutant granulomas in *csf1r* mutant versus wild-type fish, we observed more necrosis in *csf1r* mutants (Figure 4A). These gradations in necrosis were reflected in the bacterial burdens (Figure 4B). Together these results reveal the inherent connection between macrophage demand and supply, even when these two quantities appear to be independently regulated at this juncture. In addition, the finding that the ESX-1 mutant bacteria were rendered more virulent by Csf-1r deficiency has relevance to mycobacterial susceptibility of humans with myeloid deficiencies, as discussed in the following section.

Macrophage Depletion Caused by *irf8* Deficiency Confers Susceptibility

Mutations in hematopoietic transcription factors such as *PU.1* and *IRF8* are also associated with numerical deficits in macrophages (DeKoter et al., 2007; Terry and Miller, 2014). We were particularly interested in IRF8 deficiency, because even a modest reduction in monocytes is associated with human susceptibility to TB as well as to the ESX-1-deficient vaccine strain BCG (Crosslin et al., 2013; Ding et al., 2012; Hambleton et al., 2011).

irf8 homozygous mutant (*irf8*^{st95} and *irf8*^{st96}) zebrafish larvae have a near complete lack of macrophages without any obvious developmental abnormalities (Shiau et al., 2015). We showed that the mutant larvae had the expected dramatic early increase in bacterial burdens with cording that is associated with bacteria being in an extracellular niche from the start of infection (Figures 5A and 5B and data not shown) (Clay et al., 2007). To recapitulate the reduced macrophage pool of human IRF8 deficiency (rather than the complete absence of macrophages), we used a morpholino to knock down *irf8* expression (Li et al., 2011). After confirming that we could achieve the expected 95% macrophage depletion using the previously reported concentration, we titrated down the concentration of morpholino to 0.2 mM, so as to achieve partial macrophage depletion (Figure 5C). At this concentration, we found an infection phenotype similar to that of *csf1r* deficiency: increased bacterial cording and growth following upon granuloma formation (Figures 5D and 5E). These observations extend our findings with *csf1r* deficiency to those of other myeloid growth factors and suggest a mechanism whereby human IRF8 deficiency causes susceptibility to virulent mycobacteria as well as to the attenuated BCG vaccine strain (Ding et al., 2012; Hambleton et al., 2011).

Increasing Macrophage Numbers in Wild-Type Animals Delays Granuloma Necrosis

Our model would predict that increasing macrophage supply above wild-type should delay granuloma necrosis. To increase macrophage numbers, we injected mRNA encoding the Csf-1r ligand Csf-1a into *mpeg1:tdTomato* embryos (Figure 6A). This single administration increased macrophage numbers in zebrafish larvae, similar to the effect of recombinant CSF-1 protein in adult mice, rats, nonhuman primates, and humans (Hume and MacDonald, 2012). By 2 dpf, macrophages were increased throughout the body, including a 175% increase in the CHT, with neutrophil numbers being unchanged by 3 dpf (Figures 6A and 6B and data not shown). After infection, these animals had a 41% reduction in bacterial cording by 7 dpi (Figures 6C and 6D). We confirmed that the beneficial effect of Csf-1a was mediated specifically through Csf-1r signaling by showing that *csf1a* mRNA injection into *csf1r* mutants did not reduce cording (Figures 6C and 6D). Thus, increasing macrophage supply above wild-type promotes resistance. The therapeutic potential of this finding would be predicated upon being able to ameliorate an ongoing infection by increasing macrophage numbers, leading us to the next experiments.

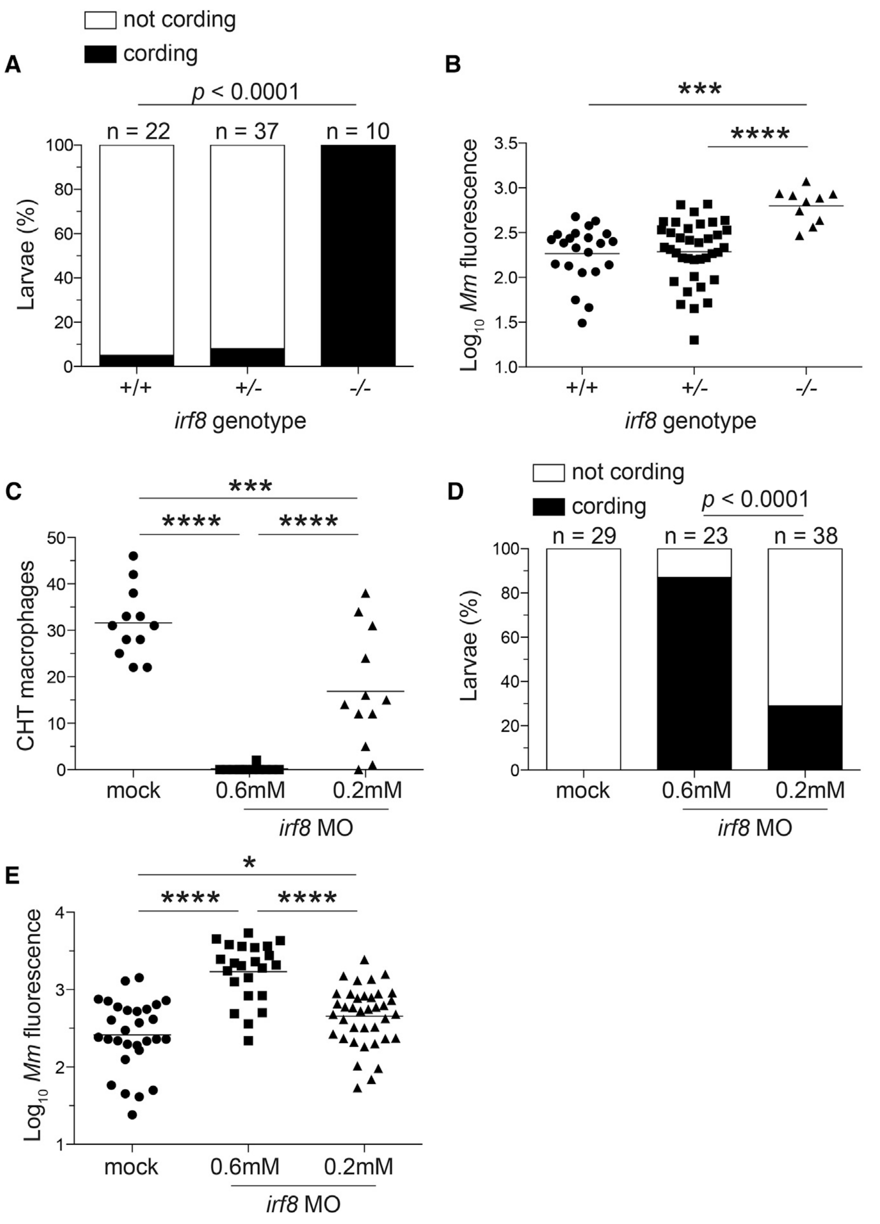

Figure 5. Macrophage Depletion Caused by *irf8* Deficiency Promotes Granuloma Necrosis

(A–E) Larvae from an $irf8^{st96/+}$ intercross were infected via the caudal vein with ~200 tdTomato-expressing *M. marinum*. (A) Percentage of larvae with cording phenotype and (B) bacterial loads 3 dpi. (C) Macrophage numbers in *irf8* morpholino (MO)- and mock-injected *mpeg1:YFP* larvae 2 dpf. (D) Bacterial cording and (E) bacterial burdens in *irf8* and mock morphants 3 dpi with ~300 tdTomato-expressing *M. marinum* injected via the caudal vein. (B, C, E, and G) Each symbol represents individual larvae, and horizontal lines indicate means. Statistical significance was determined by Fisher's exact test (A and D) or one-way ANOVA with Tukey's post-test (B, C, and E). Data are representative of three (A–D) experiments.

Restoring Macrophage Supply during Ongoing Infection Delays Granuloma Necrosis

To replenish macrophage numbers after granulomas had formed, we used a zebrafish line bearing a temperature-sensitive *csf1r* allele (*csf1ra*$^{ut.r4e174A}$, abbreviated here as *csf1r*^{ts}) (Parichy and Turner, 2003). Csf-1r signaling is preserved when *csf1r*$^{ts/ts}$ homozygous mutants or *csf1r*$^{ts/-}$ compound heterozygotes are raised at 24°C but abolished when they are raised at 33°C, as indicated by differences in xanthophore development (which is Csf-1r dependent) at the two temperatures (Parichy and Turner, 2003). We used this line to ask if restoring macrophage supply by shifting infected animals to the permissive temperature soon after granulomas formed could mitigate susceptibility. Addressing this question rigorously required the use of three genotypes and two rearing temperatures: the *csf1r*$^{ts/+}$ heterozygote, which should be macrophage-sufficient at both 33°C and 24°C; the *csf1r*$^{-/-}$ homozygote, which should be macrophage-deficient at both 33°C and 24°C; and the *csf1r*$^{ts/-}$ compound heterozygote, which should be macrophage-deficient at 33°C yet macrophage-sufficient when shifted to 24°C. We enumerated macrophages in larvae from the three lines grown at 33°C from 0 to 7 dpf or from 0 to 4 dpf, followed by a shift to 24°C through 7 dpf (Figure 7A). By 4 dpf, *csf1r*$^{-/-}$ and *csf1r*$^{ts/-}$ larvae raised at 33°C had 55% fewer macrophages than *csf1r*$^{ts/+}$ controls at both 4 and 7 dpf (Figure 6B). In contrast, *csf1r*$^{ts/-}$ larvae that were shifted to 24°C at 4 dpf had 33% fewer macrophages than *csf1r*$^{ts/+}$ controls at 7 dpf and double those of *csf1r*$^{-/-}$ larvae (Figure 7B). Thus, reinstating Csf-1r signaling during larval development had partially restored macrophage numbers within 3 days.

In the same experiment, we asked if restoring macrophage numbers during the cellular phase of the granuloma could delay its necrosis. We infected cohorts from the three lines at 2 dpf and reared them under the same two temperature conditions as their uninfected siblings (Figure 7A). Necrosis in *csf1r*$^{ts/-}$ larvae was higher than in wild-type animals at 33°C but not at 24°C (Figure 7C). In contrast, as expected, *csf1r*$^{-/-}$ animals had increased necrosis over wild-type at both temperatures (Figure 7C). When we assessed bacterial burdens, we saw the expected reduction at 24°C compared to 33°C in all genotypes, a result that was consistent with the substantial reduction in *M. marinum* growth rate at the lower temperature (Clark and Shepard, 1963). Despite this overall decrease, *csf1r*$^{-/-}$ animals still had significantly higher bacterial burdens than wild-type at the lower temperature (Figure 7D). However, in the *csf1r*$^{ts/-}$ larvae, the bacterial burdens were significantly higher than wild-type at 33°C but not at

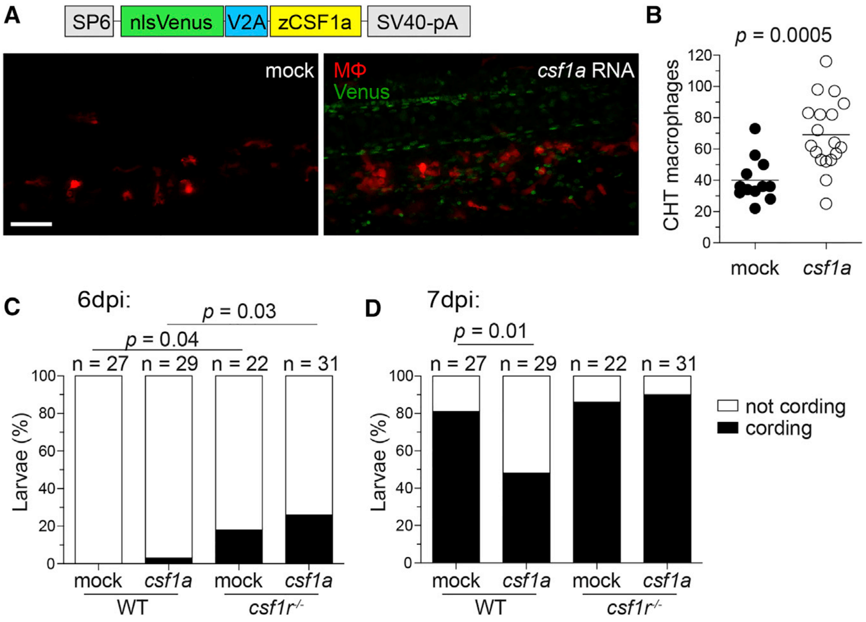

Figure 6. Increasing Macrophage Supply in Wild-Type Zebrafish Delays Granuloma Necrosis

(A–D) One-cell stage WT *mpeg1:tdTomato* embryos were injected with vehicle (mock) or ~2 nl of 200 ng/μl in vitro-transcribed *csf1a* RNA. (A) Schematic of *csf1a* overexpression construct and maximum intensity projections of the caudal hematopoietic tissue of 2-day-old larvae. Scale bar, 100 μm. (B) Number of macrophages in the CHT of 2 dpf larvae. Horizontal lines depict means. Percentage of cording in WT or *csf1r* mutant larvae 6 dpi (C) or 7 dpi (D) with ~200 *M. marinum* expressing tdKatushka2. Statistical significance determined by two-tailed unpaired Student's t test (B) or Fisher's exact test (C and D).

24°C, likely a consequence of reduced granuloma necrosis at the lower temperature (Figure 7D). Our finding that increasing macrophage supply after granulomas have formed can mitigate susceptibility may suggest the therapeutic potential of myeloid growth factors for TB.

DISCUSSION

This work suggests that tuberculous granuloma dynamics are best understood in the context of two discrete stages of granuloma development: an initial stage of cellular growth and a subsequent stage of necrosis. Both stages can promote mycobacterial growth, although the unfettered extracellular growth of the second, necrotic phase can greatly outstrip the intracellular growth of the first phase.

Macrophage supply and demand are key determinants of granuloma fate (Figure S5). The first cellular stage is dominated by macrophage demand and persists until macrophage supply becomes limiting, at which time the granuloma enters the second necrotic stage. Under the infection conditions studied here, macrophage supply and demand are determined independently of each other. However, an increased macrophage demand will deplete macrophage supply sooner. Therefore, macrophage demand in the first phase can influence the tempo of the granuloma's transition to the necrotic phase. Accordingly, reducing macrophage demand (e.g., ESX-1 mutant infection) not only delays necrosis in wild-type animals but also greatly curtails necrosis even under conditions of reduced macrophage supply (Figure S5).

Conversely, increasing macrophage supply offsets the susceptibility of wild-type animals (Figure S5). This latter result is important because it suggests that macrophage supply can be ultimately limiting even in wild-type animals. Because the findings that support this conclusion were made in larvae in a limited time course, it might be argued that it cannot be extrapolated to adult infection, which occurs in the context of a larger myeloid pool and adequate time for sustained monocyte recruitment to come into play (Shi and Pamer, 2011). Two lines of evidence support the possibility that the macrophage supply becomes ultimately limiting in adults: (1) macrophage turnover is high even in mature tuberculous granulomas of mice, frogs, and zebrafish (Cosma et al., 2004, 2008; Dannenberg, 1993, 2003) and (2) ESX-1 mutant granulomas of adult zebrafish have much less necrosis than wild-type ones, in addition to being less well formed (Swaim et al., 2006). These previous findings had been interpreted to suggest that the ESX-1 locus influences both granuloma cellularity and necrosis, possibly by distinct mechanisms (Swaim et al., 2006). The present work provides a unifying mechanistic explanation for both phenotypes by implicating the reduced macrophage demand of the mutant in both.

Our findings may explain the susceptibility to mycobacteriosis of individuals with genetic *IRF8* variants that produce deficiencies in macrophage numbers, e.g., loss-of-function *IRF8* mutations. Humans with rare variants in the DNA binding domain of *IRF8* had fewer monocytes and got disseminated BCG infection as infants soon after vaccination (Hambleton et al., 2011). Additionally, common variants in *IRF8* are associated with monocyte abundance and with susceptibility to virulent TB, although different variants were examined in the two studies (Crosslin et al., 2013; Ding et al., 2012). Based on findings in *Irf8*- and *Csf1*-deficient mice, it has been suggested that these deficiencies may cause susceptibility through poor antigen presentation leading to defective T cell immunity (Hambleton et al., 2011; Teitelbaum et al., 1999; Turcotte et al., 2005). Our work suggests that the macrophage deficiencies associated with these states can be directly responsible for susceptibility. Moreover, our finding that ESX-1 mutants eventually cause granuloma breakdown in macrophage-deficient hosts mirrors BCG-associated disseminated infection in IRF8-deficient humans (Hambleton et al., 2011). Similarly, a macrophage supply deficit may also explain the susceptibility of severely monocytopenic *GATA2*-deficient individuals to *Mycobacterium avium* complex (Dickinson et al., 2014; Hsu et al., 2011; Vinh et al., 2010).

In terms of TB pathogenesis, our findings reveal yet another route to macrophage necrosis. Previously, we had identified macrophage necrosis caused by both TNF deficiency and

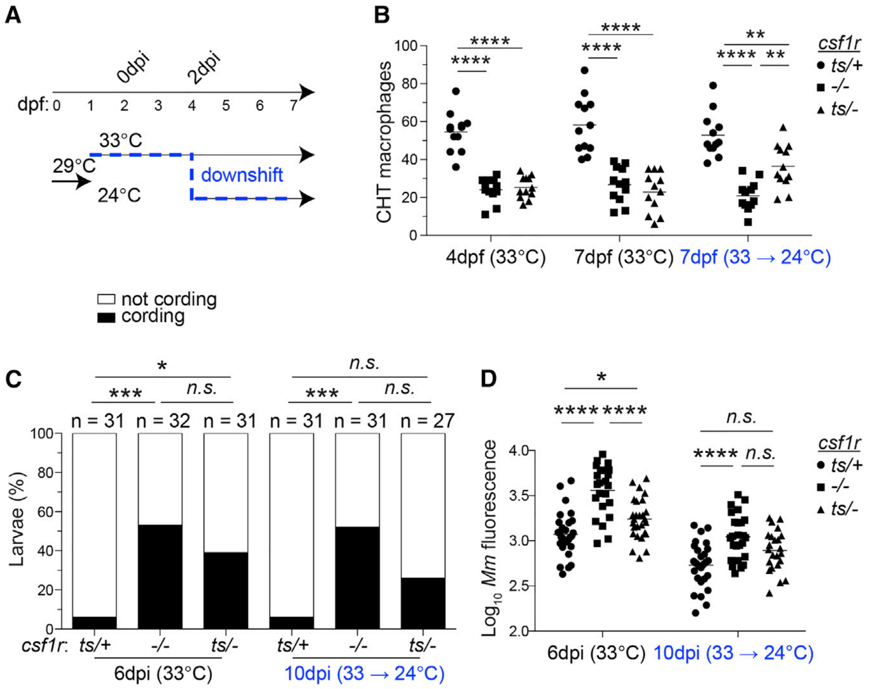

Figure 7. Restoring CSF-1R Signaling during an Ongoing Infection Delays Granuloma Necrosis

(A) Schematic of temperature-shifting experiment.
(B–D) Macrophage numbers (B), bacterial cording (C), and bacterial burdens (D) in phenotypically WT heterozygotes ($csf1r^{ts/+}$), *csf1r* null homozygous mutants ($csf1r^{-/-}$), and temperature-sensitive heterozygotes ($csf1r^{ts/-}$). Horizontal lines indicate means. Statistical significance was determined by one-way ANOVA with Tukey's post-test (B and D) or Fisher's exact test, correcting for multiple comparisons by multiplying p values obtained in pairwise comparisons by the number of groups in each experimental condition (C).

excess and had identified common human variants in the Leukotriene A4 Hydrolase gene that promoted susceptibility, presumably by dysregulation of TNF (Clay et al., 2008; Roca and Ramakrishnan, 2013; Tobin et al., 2012). We now find that genetic defects in macrophage development can produce the same phenotypic compromise in granuloma integrity by reducing the pool of macrophages that would replace those dying in the granuloma. We envision that any genetic or induced defect that reduces the availability of functional macrophages (e.g., reduced macrophage survival) could exacerbate granuloma necrosis.

In summary, our findings suggest that maintaining an adequate macrophage supply promotes resistance by preserving granuloma integrity. Even in the face of pathological, mycobacterium-accelerated macrophage demand, sustaining the macrophage pool benefits the host by permitting the bacteria only intracellular rather than extracellular growth. Therefore, boosting macrophage supply with myeloid growth factors may be a host-targeting therapy for TB. Indeed, our experiments show that increasing the myeloid pool even after granulomas have formed can reduce their necrosis. However, these proof-of-concept experiments could only assess the effect of macrophage replenishment during the early cellular phase of the granuloma. It is possible that once a granuloma has become highly necrotic (as in patients with advanced TB), the corded extracellular bacteria therein can no longer be phagocytosed by new macrophages (Bernut et al., 2014; Pagán and Ramakrishnan, 2014). This caveat notwithstanding, myeloid growth factor therapies may be worth trying, particularly in extensively drug-resistant TB, where antimicrobial therapy alone offers little hope for cure (Wong et al., 2013).

EXPERIMENTAL PROCEDURES

Bacterial Strains

M. marinum M strain (ATCC #BAA-535) and its mutant derivatives ΔESX-1 (ΔRD1) and Δ*erp* expressing tdTomato or tdKatushka2 under control of the *msp12* promoter (Cosma et al., 2006a; Takaki et al., 2013; Volkman et al., 2004) were grown under hygromycin (Mediatech) or kanamycin (Sigma) selection in 7H9 Middlebrook's medium (Difco) supplemented with oleic acid, albumin, dextrose, and Tween-80 (Sigma) (Takaki et al., 2013).

Zebrafish Husbandry and Infections

Zebrafish husbandry and experiments were conducted in compliance with guidelines from the UK Home Office and the U.S. National Institutes of Health and approved by the University of Washington Institutional Animal Care and Use Committee. The Tg(*mpeg1:Brainbow*)^{w201} line was generated by cloning the *Brainbow 1.0L* cassette (Livet et al., 2007) (Addgene) into a Tol2 plasmid containing the zebrafish *mpeg1* promoter (Ellett et al., 2011). The *mpeg1:Brainbow* plasmid was then injected along with transposase mRNA into one- to two-cell-stage embryos of the wild-type AB strain (Zebrafish International Resource Center) as previously described (Suster et al., 2011). Putative founders were identified by tdTomato expression in macrophages and crossed to wild-type AB zebrafish. Transgenic lines were identified in the next generation and kept on the AB strain. Wild-type AB strain, $csf1ra^{j4blue}$ (Parichy et al., 2000), $csf1ra^{ut.r4e174A}$ (Parichy and Turner, 2003), $irf8^{st95}$ and $irf8^{st96}$ (Shiau et al., 2015), Tg(*mpeg1:Gal4FF*)^{gl25} × Tg(*UAS:E1bKaede*)^{s1999t} (Ellett et al., 2011), Tg(*mpeg1:YFP*)^{w200} (Roca and Ramakrishnan, 2013), Tg(*mpeg1:Brainbow*)^{w201}, and Tg(*lysC:EGFP*)^{nz117} (Hall et al., 2007) larvae were infected via the caudal vein or hindbrain ventricle at 2 dpf as previously described (Takaki et al., 2013). Adults were infected intraperitoneally as described previously (Cosma et al., 2006b). Homozygous $csf1ra^{j4blue}$ larvae were identified from phenotypically wild-type siblings by their xanthophore deficit (Parichy et al., 2000). $irf8^{st95}$ and $irf8^{st96}$ lines were genotyped by high-resolution melt analysis (Garritano et al., 2009) of PCR products generated with the following primers: Forward, 5′-TGGATGCCGTGAGTATGTAC-3′ and Rev, 5′-CCTCCCACTG CAGTCCATTA-3′ on a CFX Connect thermocycler (BioRad).

Construction of *Venus-V2A-csf1a* Plasmid and In Vitro Transcription

Danio rerio csf1a was cloned into a Gateway middle entry vector encoding nuclear-localized Venus and a viral 2A peptide cleavage sequence (Provost et al., 2007) and subsequently assembled into a Gateway destination vector to produce *pCMV:nlsVenus-V2A-csf1a* (gift from D. Parichy). In vitro transcription was performed with mMessage/mMachine SP6 kit (Life Technologies).

Morpholino, RNA, and Liposome Injections

irf8 splice-blocking morpholino (5′-AATGTTTCGCTTACTTTGAAAATGG-3′) (Li et al., 2011) (Gene Tools) and in vitro-transcribed *csf1a* mRNA were diluted in a 1× Buffer Tango (Thermo Scientific) containing 2% phenol red sodium salt solution (Sigma) and injected into the yolk of one- to two-cell-stage embryos in ~2 nl (Tobin et al., 2012). Lipo-PBS and lipo-clodronate (http://clodronateliposomes.org) (van Rooijen et al., 1996) were diluted in PBS and injected into 2-dpf-old larvae in ~10 nl via the caudal vein.

Quantitative RT-PCR

Quantitative RT-PCR was performed as described (Clay et al., 2007). Total RNA from batches of ~30 embryos per biological replicate was isolated with TRIzol Reagent (Life Technologies) and used to synthesize cDNA with Superscript II reverse transcriptase and oligo DT primers (Invitrogen). Quantitative RT-PCR assays were performed with SYBR green PCR Master Mix (Applied Biosystems) on an ABI Prism 7300 Real-Time PCR System (Applied Biosystems). Each biological replicate was run in triplicate, and average values were plotted. Data were normalized to *bactin* for ΔΔCt analysis.

Sudan Black Staining

Sudan Black staining was performed as described (Le Guyader et al., 2008). Embryos were fixed in 4% methanol-free paraformaldehyde at 4°C overnight. On the following day, embryos were washed in PBS and stained with Sudan Black B Staining Reagent (Sigma) at room temperature for 20 min. Stained embryos were extensively washed with 70% ethanol and then gradually rehydrated to PBS containing 0.1% Tween-20.

Histology

Histology was performed as described (Swaim et al., 2006). Euthanized fish were fixed in Dietrich's fixative for 72 hr, transferred into 70% ethanol, and sent to Histo-Tec Laboratories for processing. Fish were embedded in paraffin and sectioned along the midline. Serial sagittal sections of 7 μm were stained with hematoxylin and eosin or modified Ziehl-Neelsen stain.

Microscopy

Fluorescence microscopy was performed as described (Takaki et al., 2013; Yang et al., 2012). Quantification of bacterial burdens, assessments of mycobacterial cording, and enumeration of neutrophils were performed with a Nikon Eclipse Ti-E inverted microscope fitted with 2×, 10×, and 20× objectives. Enumeration of macrophages, assessments of intracellular bacterial growth, measurements of granuloma diameter, and evaluation of histological sections were performed on a Nikon Eclipse E600 upright microscope fitted with 10× and 20× objectives. For laser scanning confocal microscopy, larvae were anesthetized in *N*-phenylthiourea (PTU)-supplemented fish water containing 0.025% Tricaine and embedded in 1.5% low-melting-point agarose on optical bottom plates or dishes (MatTek Corporation). A Nikon A1 confocal microscope with a 20× Plan Apo 0.75 NA objective was used to generate 40 μm z stacks consisting of 1.3–2 μm optical sections. The galvano scanner was used for all static imaging and for time-lapse imaging of the CHT. Time-lapse images were taken at 5 min intervals for 8 hr. Data were acquired with NIS Elements (Nikon). Macrophage tracks were generated using the 3D tracking feature of Imaris (Bitplane Scientific Software).

Flow Cytometry

Splenocyte single-cell suspensions of 4- to 5-month-old zebrafish were prepared by dissociating the tissue in flow cytometry buffer (1× PBS, 2% FCS, and 1 mM EDTA) and filtering it through a 70 μm cell strainer (BD Biosciences). Liver samples were digested in 1× PBS supplemented with DNase I and type I/II collagenases for 15 min at 32°C. Cells were then stained with Alexa Fluor 594-conjugated Peanut Agglutinin (PNA) (Life Technologies) for 30 min at 4°C to enrich for myeloid cells, as previously described (Lugo-Villarino et al., 2010). Samples were resuspended in flow cytometry buffer containing 0.05 μg/ml 4′,6-Diamidino-2-phenylindole dihydrochloride (DAPI) (Sigma) for dead cell exclusion and run on an LSRII flow cytometer (BD Biosciences). Absolute numbers of cells were calculated with AccuCheck Counting Beads (Life Technologies) (Moon et al., 2009). Samples were analyzed with FlowJo (TreeStar).

Statistical Analyses

Statistical analyses were performed on Prism (GraphPad). Not significant, $p \geq 0.05$; * $p < 0.05$; ** $p < 0.01$; *** $p < 0.001$; **** $p < 0.0001$.

SUPPLEMENTAL INFORMATION

Supplemental Information includes five figures and two movies and can be found with this article online at http://dx.doi.org/10.1016/j.chom.2015.06.008.

AUTHOR CONTRIBUTIONS

A.J.P., C-T.Y., and L.R. designed experiments and analyzed the data. A.J.P. and C-T.Y. performed experiments. J.C. performed adult fish experiments in Figure 2. L.E.S. designed, performed, and analyzed the larval survival experiment in Figure 1C. F.E. and G.J.L. provided the *mpeg1:Gal4FF* Tol2 plasmid and *mpeg1:Gal4FF; UAS:E1bKaede* fish prior to their publication. A.J.P. and C-T.Y. prepared the figures, and A.J.P. and L.R. wrote the paper with input from C-T.Y.

ACKNOWLEDGMENTS

We thank B. Cormack, S. Falkow, and S. Levitte for discussions; B. Cormack, J.M. Davis, S. Falkow, S. Levitte, D. Parichy, and D. Tobin for manuscript review; P. Edelstein for help with statistical analysis; D. Parichy and W. Talbot for zebrafish lines or constructs; G. Dougan for providing transitional laboratory facilities at the Sanger Institute; N. Goodwin for zebrafish husbandry; D. Prunkard and the University of Washington Department of Pathology's Flow Cytometry Core facility for assistance with flow cytometry experiments; and L. Galitan and R.E. Hernandez for assistance with adult fish experiments. This work was funded by grants from the National Institutes of Health (T32-AI055396, A.J.P.; A154503 and A136396, L.R.) and the National Health and Medical Research Council (637394, 1044754, and 1069284, G.J.L.), a postdoctoral fellowship from the Taiwan National Science Council (NSC97-2917-I-564-109, C-T.Y.), and an Australian Postgraduate Award and Walter and Eliza Hall Institute of Medical Research Edith Moffatt Scholarship (F.E.). The Australian Regenerative Medicine Institute is supported by funds from the State Government of Victoria and the Australian Federal Government. L.R. is a recipient of the NIH Director's Pioneer Award and a Wellcome Trust Principal Research Fellowship.

Received: March 29, 2015
Revised: June 1, 2015
Accepted: June 19, 2015
Published: July 8, 2015

REFERENCES

Bernut, A., Herrmann, J.L., Kissa, K., Dubremetz, J.F., Gaillard, J.L., Lutfalla, G., and Kremer, L. (2014). Mycobacterium abscessus cording prevents phagocytosis and promotes abscess formation. Proc. Natl. Acad. Sci. USA *111*, E943–E952.

Boocock, C.A., Jones, G.E., Stanley, E.R., and Pollard, J.W. (1989). Colony-stimulating factor-1 induces rapid behavioural responses in the mouse macrophage cell line, BAC1.2F5. J. Cell Sci. *93*, 447–456.

Cambier, C.J., Falkow, S., and Ramakrishnan, L. (2014). Host evasion and exploitation schemes of Mycobacterium tuberculosis. Cell *159*, 1497–1509.

Cecchini, M.G., Dominguez, M.G., Mocci, S., Wetterwald, A., Felix, R., Fleisch, H., Chisholm, O., Hofstetter, W., Pollard, J.W., and Stanley, E.R. (1994). Role of colony stimulating factor-1 in the establishment and regulation of tissue macrophages during postnatal development of the mouse. Development *120*, 1357–1372.

Clark, H.F., and Shepard, C.C. (1963). Effect of Environmental Temperatures on Infection with Mycobacterium Marinum (Balnei) of Mice and a Number of Poikilothermic Species. J. Bacteriol. *86*, 1057–1069.

Clay, H., Davis, J.M., Beery, D., Huttenlocher, A., Lyons, S.E., and Ramakrishnan, L. (2007). Dichotomous role of the macrophage in early Mycobacterium marinum infection of the zebrafish. Cell Host Microbe *2*, 29–39.

Clay, H., Volkman, H.E., and Ramakrishnan, L. (2008). Tumor necrosis factor signaling mediates resistance to mycobacteria by inhibiting bacterial growth and macrophage death. Immunity *29*, 283–294.

Clements, W.K., and Traver, D. (2013). Signalling pathways that control vertebrate haematopoietic stem cell specification. Nat. Rev. Immunol. *13*, 336–348.

Cosma, C.L., Humbert, O., and Ramakrishnan, L. (2004). Superinfecting mycobacteria home to established tuberculous granulomas. Nat. Immunol. *5*, 828–835.

Cosma, C.L., Klein, K., Kim, R., Beery, D., and Ramakrishnan, L. (2006a). Mycobacterium marinum Erp is a virulence determinant required for cell wall integrity and intracellular survival. Infect. Immun. *74*, 3125–3133.

Cosma, C.L., Swaim, L.E., Volkman, H., Ramakrishnan, L., and Davis, J.M. (2006b). Zebrafish and frog models of Mycobacterium marinum infection. Curr. Protoc. Microbiol. *Chapter 10*, 2, http://dx.doi.org/10.1002/0471729256.mc10b02s3.

Cosma, C.L., Humbert, O., Sherman, D.R., and Ramakrishnan, L. (2008). Trafficking of superinfecting Mycobacterium organisms into established granulomas occurs in mammals and is independent of the Erp and ESX-1 mycobacterial virulence loci. J. Infect. Dis. *198*, 1851–1855.

Crosslin, D.R., McDavid, A., Weston, N., Zheng, X., Hart, E., de Andrade, M., Kullo, I.J., McCarty, C.A., Doheny, K.F., Pugh, E., et al.; CHARGE Hematology Working Group; electronic Medical Records and Genomics (eMERGE) Network (2013). Genetic variation associated with circulating monocyte count in the eMERGE Network. Hum. Mol. Genet. *22*, 2119–2127.

Dai, X.M., Ryan, G.R., Hapel, A.J., Dominguez, M.G., Russell, R.G., Kapp, S., Sylvestre, V., and Stanley, E.R. (2002). Targeted disruption of the mouse colony-stimulating factor 1 receptor gene results in osteopetrosis, mononuclear phagocyte deficiency, increased primitive progenitor cell frequencies, and reproductive defects. Blood *99*, 111–120.

Dannenberg, A.M., Jr. (1993). Immunopathogenesis of pulmonary tuberculosis. Hosp. Pract. (Off. Ed) *28*, 51–58.

Dannenberg, A.M., Jr. (2003). Macrophage turnover, division and activation within developing, peak and "healed" tuberculous lesions produced in rabbits by BCG. Tuberculosis (Edinb.) *83*, 251–260.

Davis, J.M., and Ramakrishnan, L. (2009). The role of the granuloma in expansion and dissemination of early tuberculous infection. Cell *136*, 37–49.

Davis, J.M., Clay, H., Lewis, J.L., Ghori, N., Herbomel, P., and Ramakrishnan, L. (2002). Real-time visualization of mycobacterium-macrophage interactions leading to initiation of granuloma formation in zebrafish embryos. Immunity *17*, 693–702.

DeKoter, R.P., Kamath, M.B., and Houston, I.B. (2007). Analysis of concentration-dependent functions of PU.1 in hematopoiesis using mouse models. Blood Cells Mol. Dis. *39*, 316–320.

Dickinson, R.E., Milne, P., Jardine, L., Zandi, S., Swierczek, S.I., McGovern, N., Cookson, S., Ferozepurwalla, Z., Langridge, A., Pagan, S., et al. (2014). The evolution of cellular deficiency in GATA2 mutation. Blood *123*, 863–874.

Ding, S., Jiang, T., He, J., Qin, B., Lin, S., and Li, L. (2012). Tagging single nucleotide polymorphisms in the IRF1 and IRF8 genes and tuberculosis susceptibility. PLoS ONE *7*, e42104.

Ellett, F., Pase, L., Hayman, J.W., Andrianopoulos, A., and Lieschke, G.J. (2011). mpeg1 promoter transgenes direct macrophage-lineage expression in zebrafish. Blood *117*, e49–e56.

Garritano, S., Gemignani, F., Voegele, C., Nguyen-Dumont, T., Le Calvez-Kelm, F., De Silva, D., Lesueur, F., Landi, S., and Tavtigian, S.V. (2009). Determining the effectiveness of High Resolution Melting analysis for SNP genotyping and mutation scanning at the TP53 locus. BMC Genet. *10*, 5.

Ginhoux, F., and Jung, S. (2014). Monocytes and macrophages: developmental pathways and tissue homeostasis. Nat. Rev. Immunol. *14*, 392–404.

Ginhoux, F., Greter, M., Leboeuf, M., Nandi, S., See, P., Gokhan, S., Mehler, M.F., Conway, S.J., Ng, L.G., Stanley, E.R., et al. (2010). Fate mapping analysis reveals that adult microglia derive from primitive macrophages. Science *330*, 841–845.

Hall, C., Flores, M.V., Storm, T., Crosier, K., and Crosier, P. (2007). The zebrafish lysozyme C promoter drives myeloid-specific expression in transgenic fish. BMC Dev. Biol. *7*, 42.

Hambleton, S., Salem, S., Bustamante, J., Bigley, V., Boisson-Dupuis, S., Azevedo, J., Fortin, A., Haniffa, M., Ceron-Gutierrez, L., Bacon, C.M., et al. (2011). IRF8 mutations and human dendritic-cell immunodeficiency. N. Engl. J. Med. *365*, 127–138.

Herbomel, P., Thisse, B., and Thisse, C. (1999). Ontogeny and behaviour of early macrophages in the zebrafish embryo. Development *126*, 3735–3745.

Herbomel, P., Thisse, B., and Thisse, C. (2001). Zebrafish early macrophages colonize cephalic mesenchyme and developing brain, retina, and epidermis through a M-CSF receptor-dependent invasive process. Dev. Biol. *238*, 274–288.

Hsu, A.P., Sampaio, E.P., Khan, J., Calvo, K.R., Lemieux, J.E., Patel, S.Y., Frucht, D.M., Vinh, D.C., Auth, R.D., Freeman, A.F., et al. (2011). Mutations in GATA2 are associated with the autosomal dominant and sporadic monocytopenia and mycobacterial infection (MonoMAC) syndrome. Blood *118*, 2653–2655.

Hume, D.A., and MacDonald, K.P. (2012). Therapeutic applications of macrophage colony-stimulating factor-1 (CSF-1) and antagonists of CSF-1 receptor (CSF-1R) signaling. Blood *119*, 1810–1820.

Langenau, D.M., and Zon, L.I. (2005). The zebrafish: a new model of T-cell and thymic development. Nat. Rev. Immunol. *5*, 307–317.

Le Guyader, D., Redd, M.J., Colucci-Guyon, E., Murayama, E., Kissa, K., Briolat, V., Mordelet, E., Zapata, A., Shinomiya, H., and Herbomel, P. (2008). Origins and unconventional behavior of neutrophils in developing zebrafish. Blood *111*, 132–141.

Lewis, K.N., Liao, R., Guinn, K.M., Hickey, M.J., Smith, S., Behr, M.A., and Sherman, D.R. (2003). Deletion of RD1 from Mycobacterium tuberculosis mimics bacille Calmette-Guérin attenuation. J. Infect. Dis. *187*, 117–123.

Li, L., Jin, H., Xu, J., Shi, Y., and Wen, Z. (2011). Irf8 regulates macrophage versus neutrophil fate during zebrafish primitive myelopoiesis. Blood *117*, 1359–1369.

Lieschke, G.J., Oates, A.C., Paw, B.H., Thompson, M.A., Hall, N.E., Ward, A.C., Ho, R.K., Zon, L.I., and Layton, J.E. (2002). Zebrafish SPI-1 (PU.1) marks a site of myeloid development independent of primitive erythropoiesis: implications for axial patterning. Dev. Biol. *246*, 274–295.

Livet, J., Weissman, T.A., Kang, H., Draft, R.W., Lu, J., Bennis, R.A., Sanes, J.R., and Lichtman, J.W. (2007). Transgenic strategies for combinatorial expression of fluorescent proteins in the nervous system. Nature *450*, 56–62.

Lugo-Villarino, G., Balla, K.M., Stachura, D.L., Bañuelos, K., Werneck, M.B., and Traver, D. (2010). Identification of dendritic antigen-presenting cells in the zebrafish. Proc. Natl. Acad. Sci. USA *107*, 15850–15855.

Moon, J.J., Chu, H.H., Hataye, J., Pagán, A.J., Pepper, M., McLachlan, J.B., Zell, T., and Jenkins, M.K. (2009). Tracking epitope-specific T cells. Nat. Protoc. *4*, 565–581.

Pagán, A.J., and Ramakrishnan, L. (2014). Immunity and Immunopathology in the Tuberculous Granuloma. Cold Spring Harb Perspect Med. http://dx.doi.org/10.1101/cshperspect.a018499.

Parichy, D.M., and Turner, J.M. (2003). Temporal and cellular requirements for Fms signaling during zebrafish adult pigment pattern development. Development *130*, 817–833.

Parichy, D.M., Ransom, D.G., Paw, B., Zon, L.I., and Johnson, S.L. (2000). An orthologue of the kit-related gene fms is required for development of neural crest-derived xanthophores and a subpopulation of adult melanocytes in the zebrafish, Danio rerio. Development *127*, 3031–3044.

Provost, E., Rhee, J., and Leach, S.D. (2007). Viral 2A peptides allow expression of multiple proteins from a single ORF in transgenic zebrafish embryos. Genesis *45*, 625–629.

Ramakrishnan, L. (2012). Revisiting the role of the granuloma in tuberculosis. Nat. Rev. Immunol. *12*, 352–366.

Ramakrishnan, L. (2014). The Zebrafish Guide to Tuberculosis Immunity and Treatment. Cold Spring Harb. Symp. Quant. Biol. *78*, 179–192, http://dx.doi.org/10.1101/sqb.2013.78.023283.

Roca, F.J., and Ramakrishnan, L. (2013). TNF dually mediates resistance and susceptibility to mycobacteria via mitochondrial reactive oxygen species. Cell *153*, 521–534.

Sampaio, N.G., Yu, W., Cox, D., Wyckoff, J., Condeelis, J., Stanley, E.R., and Pixley, F.J. (2011). Phosphorylation of CSF-1R Y721 mediates its association with PI3K to regulate macrophage motility and enhancement of tumor cell invasion. J. Cell Sci. *124*, 2021–2031.

Shi, C., and Pamer, E.G. (2011). Monocyte recruitment during infection and inflammation. Nat. Rev. Immunol. *11*, 762–774.

Shiau, C.E., Kaufman, Z., Meireles, A.M., and Talbot, W.S. (2015). Differential requirement for irf8 in formation of embryonic and adult macrophages in zebrafish. PLoS ONE *10*, e0117513.

Stanley, E.R., and Chitu, V. (2014). CSF-1 receptor signaling in myeloid cells. Cold Spring Harb. Perspect. Biol. *6*, a021857.

Suster, M.L., Abe, G., Schouw, A., and Kawakami, K. (2011). Transposon-mediated BAC transgenesis in zebrafish. Nat. Protoc. *6*, 1998–2021.

Swaim, L.E., Connolly, L.E., Volkman, H.E., Humbert, O., Born, D.E., and Ramakrishnan, L. (2006). Mycobacterium marinum infection of adult zebrafish causes caseating granulomatous tuberculosis and is moderated by adaptive immunity. Infect. Immun. *74*, 6108–6117.

Takaki, K., Davis, J.M., Winglee, K., and Ramakrishnan, L. (2013). Evaluation of the pathogenesis and treatment of Mycobacterium marinum infection in zebrafish. Nat. Protoc. *8*, 1114–1124.

Teitelbaum, R., Schubert, W., Gunther, L., Kress, Y., Macaluso, F., Pollard, J.W., McMurray, D.N., and Bloom, B.R. (1999). The M cell as a portal of entry to the lung for the bacterial pathogen Mycobacterium tuberculosis. Immunity *10*, 641–650.

Terry, R.L., and Miller, S.D. (2014). Molecular control of monocyte development. Cell. Immunol. *291*, 16–21.

Tobin, D.M., Vary, J.C., Jr., Ray, J.P., Walsh, G.S., Dunstan, S.J., Bang, N.D., Hagge, D.A., Khadge, S., King, M.C., Hawn, T.R., et al. (2010). The lta4h locus modulates susceptibility to mycobacterial infection in zebrafish and humans. Cell *140*, 717–730.

Tobin, D.M., Roca, F.J., Oh, S.F., McFarland, R., Vickery, T.W., Ray, J.P., Ko, D.C., Zou, Y., Bang, N.D., Chau, T.T., et al. (2012). Host genotype-specific therapies can optimize the inflammatory response to mycobacterial infections. Cell *148*, 434–446.

Turcotte, K., Gauthier, S., Tuite, A., Mullick, A., Malo, D., and Gros, P. (2005). A mutation in the Icsbp1 gene causes susceptibility to infection and a chronic myeloid leukemia-like syndrome in BXH-2 mice. J. Exp. Med. *201*, 881–890.

van Rooijen, N., Sanders, A., and van den Berg, T.K. (1996). Apoptosis of macrophages induced by liposome-mediated intracellular delivery of clodronate and propamidine. J. Immunol. Methods *193*, 93–99.

Vinh, D.C., Patel, S.Y., Uzel, G., Anderson, V.L., Freeman, A.F., Olivier, K.N., Spalding, C., Hughes, S., Pittaluga, S., Raffeld, M., et al. (2010). Autosomal dominant and sporadic monocytopenia with susceptibility to mycobacteria, fungi, papillomaviruses, and myelodysplasia. Blood *115*, 1519–1529.

Volkman, H.E., Clay, H., Beery, D., Chang, J.C., Sherman, D.R., and Ramakrishnan, L. (2004). Tuberculous granuloma formation is enhanced by a mycobacterium virulence determinant. PLoS Biol. *2*, e367.

Webb, S.E., Pollard, J.W., and Jones, G.E. (1996). Direct observation and quantification of macrophage chemoattraction to the growth factor CSF-1. J. Cell Sci. *109*, 793–803.

Wong, E.B., Cohen, K.A., and Bishai, W.R. (2013). Rising to the challenge: new therapies for tuberculosis. Trends Microbiol. *21*, 493–501.

Yang, C.T., Cambier, C.J., Davis, J.M., Hall, C.J., Crosier, P.S., and Ramakrishnan, L. (2012). Neutrophils exert protection in the early tuberculous granuloma by oxidative killing of mycobacteria phagocytosed from infected macrophages. Cell Host Microbe *12*, 301–312.

Cell Host & Microbe, Volume *18*

Supplemental Information

Myeloid Growth Factors Promote Resistance
to Mycobacterial Infection by Curtailing Granuloma
Necrosis through Macrophage Replenishment

Antonio J. Pagán, Chao-Tsung Yang, James Cameron, Laura E. Swaim, Felix Ellett, Graham J. Lieschke, and Lalita Ramakrishnan

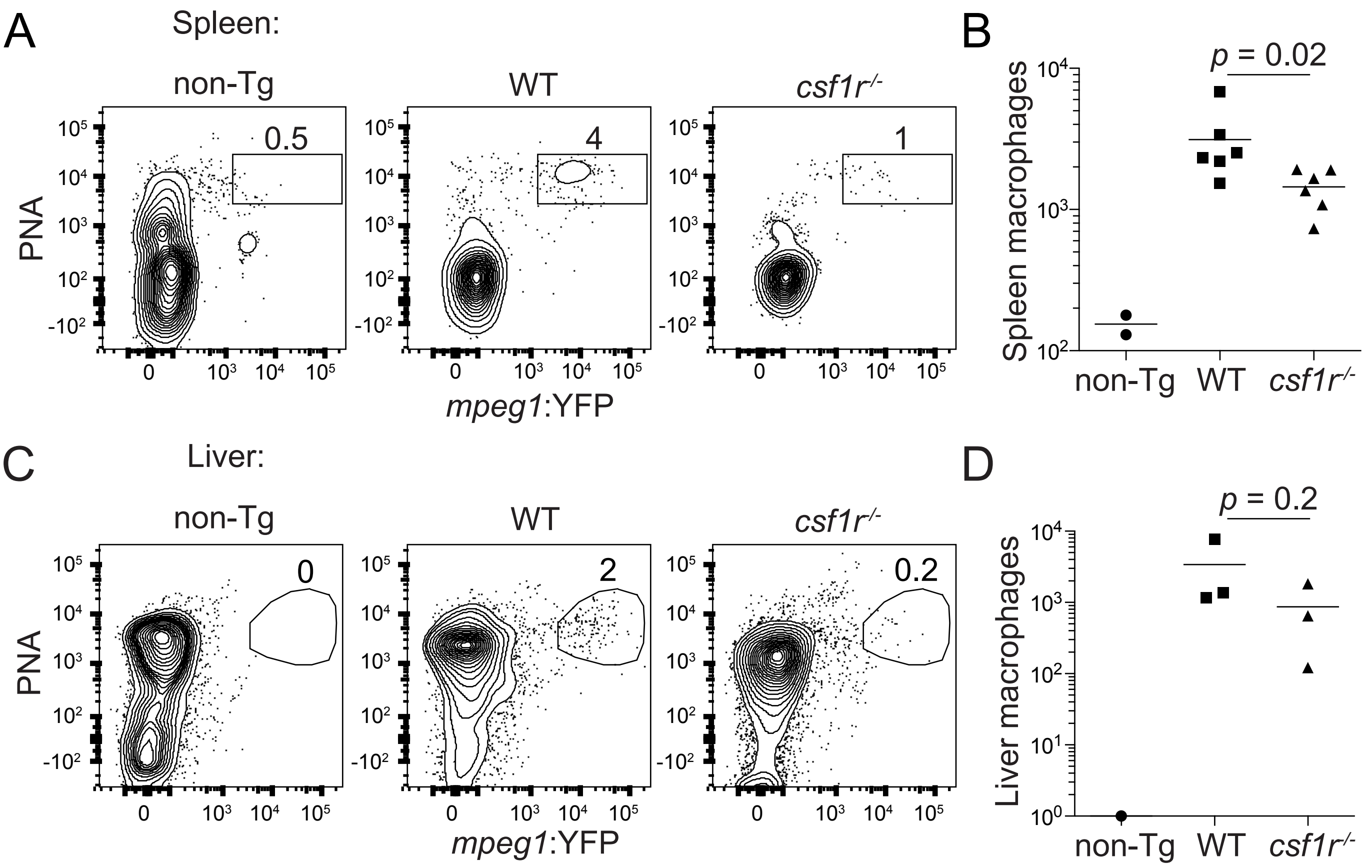

Figure S1. (Related to Figure 1). Adult csf1r mutant zebrafish have impaired macrophage development. (A) Flow cytometry plots and (B) absolute numbers of viable (DAPI$^-$) splenic macrophages in 3-4 month-old wild-type (WT) and *csf1r$^{-/-}$ mpeg1:YFP* fish. *mpeg1:YFP$^-$* WT (non-transgenic, non-Tg) fish were used as negative controls. Each symbol represents individual larvae. (C) Flow cytometry plots and (D) absolute numbers of viable (DAPI$^-$) liver macrophages in 3-4 month-old wild-type (WT) and *csf1r$^{-/-}$ mpeg1:YFP*. Numbers above gated populations indicate percentages within parent population. Data are representative of two independent experiments (A) or were pooled from two experiments (B). Statistical significance determined by two-tailed unpaired Student's t test.

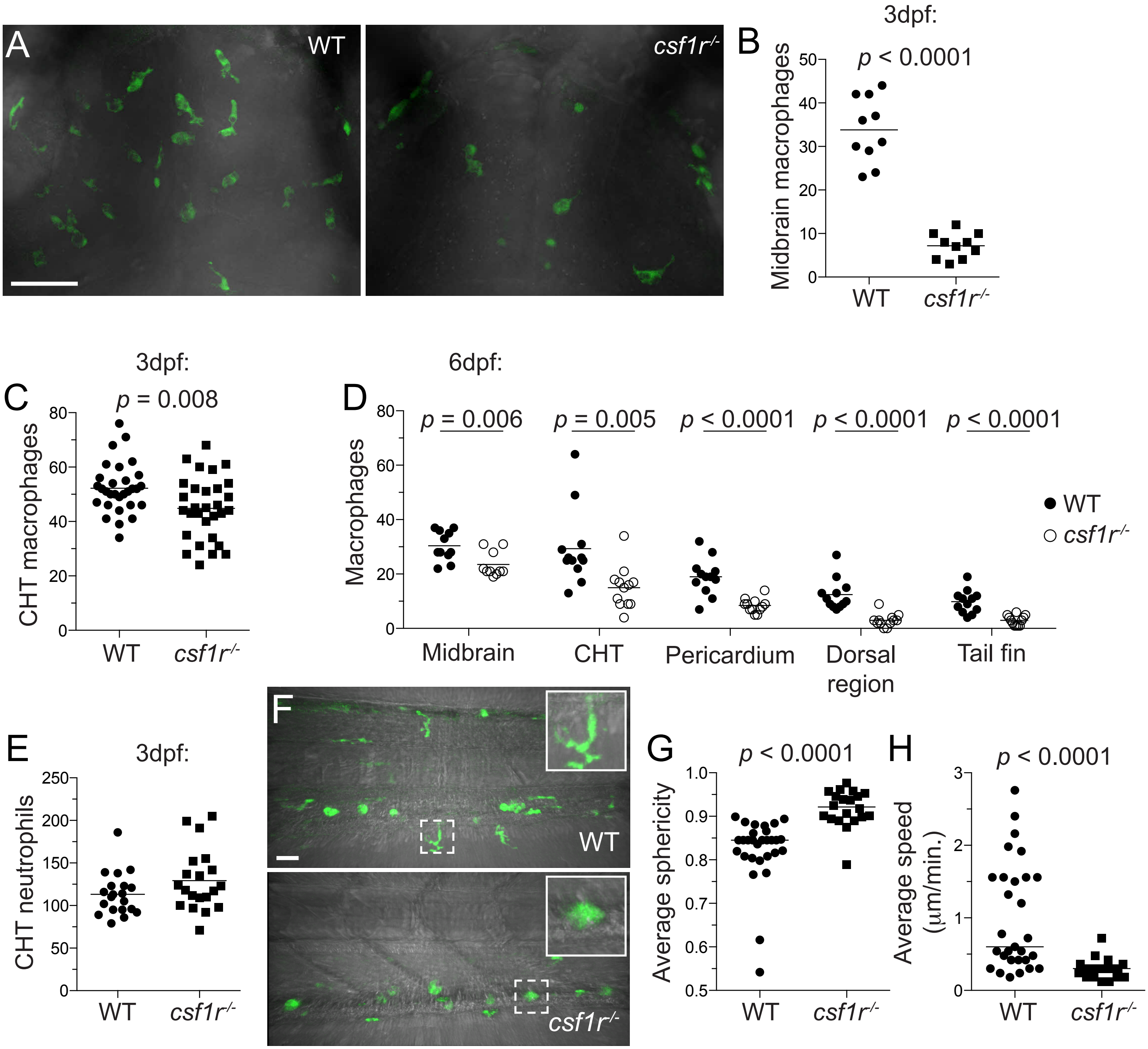

Figure S2. (Related to Figure 1). Larval csf1r mutant zebrafish have impaired macrophage development. Enumeration of larval macrophages in wild-type (WT) and *csf1r-/- mpeg1:YFP* fish. (A) Maximum intensity projections of the hindbrains of WT and *csf1r-/- mpeg1:YFP* larvae at 3dpf. Scale bars, 100μm. Number of macrophages in the (B) midbrain and (C) CHT of 3dpf fish. (D) Absolute numbers of macrophages at 6dpf. Number of neutrophils in the CHT of 3dpf fish as determined by Sudan Black staining. (B - E) Each symbol represents individual larvae. (B - D) Data are representative of at least two experiments. (F - H) Assessment of macrophage motility: (F) Representative maximum intensity projections of the CHT of WT and *csf1r-/- mpeg1:YFP* larvae at 6dpf and (G) average sphericity and (H) speed of individual macrophage tracks. Hashed boxed indicate zoomed region displayed on right corner. Scale bar, 40μm. Horizontal lines indicate means (B - E) or median values (G, H). Statistical significance determined by two-tailed unpaired Student's t test (B - E) or Mann-Whitney U test (G, H).

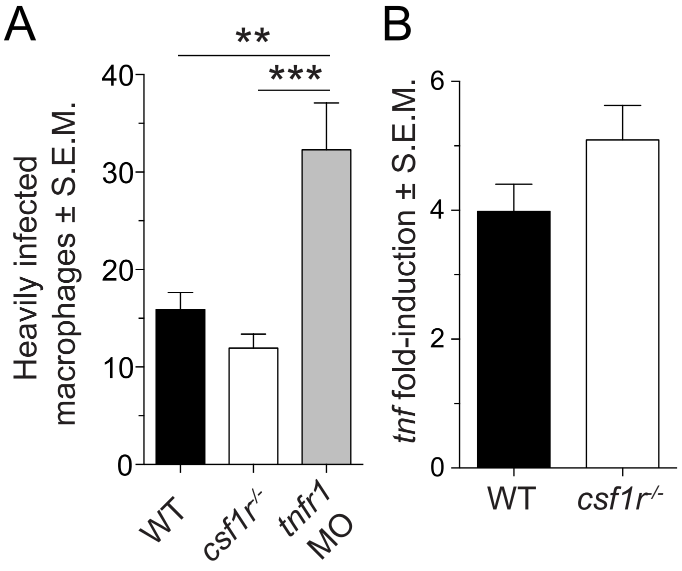

Figure S3. (Related to Figure 1). *csf1r* mutant macrophages have normal microbicidal function during mycobacterial infection. (A) Mean percentage of heavily infected macrophages in WT, *csf1r* mutant, and *tnfr1* morphant larvae 2dpi with ~186 *Δerp M. marinum*. n = 9 or 10 per group. (B) Mean induction of *tnf* expression in WT and *csf1r* mutant larvae 3dpi via the caudal vein with ~220 *M. marinum* over mock-infected, genotype-matched controls. Horizontal lines depict means. Data were pooled from three independent experiments. n = 3 biological replicates per group, each consisting of ~30 larvae. (A) One-way ANOVA with Tukey's post-test or (B) two-tailed unpaired Student's t test with Welch's correction was performed to determine statistical significance.

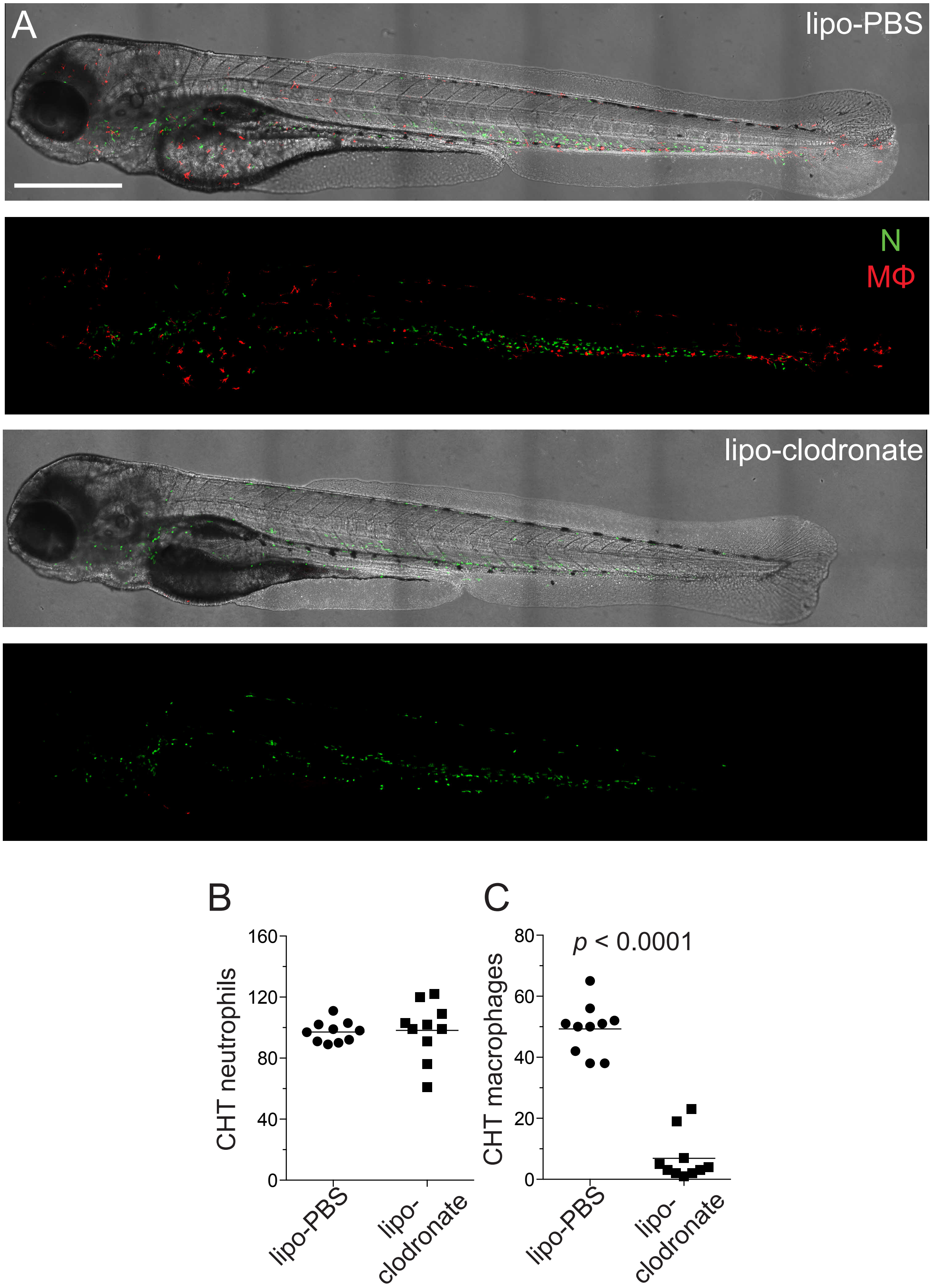

Figure S4. (Related to Figure 3). Lipo-clodronate treatment specifically depletes macrophages in zebrafish. (A) Stitched, 80µm maximum intensity projections of 4dpf *lysC:EGFP*; *mpeg1:tdTomato* larvae 40 hours after intravenous injection with 1:5 dilutions of lipo-PBS (left) or lipo-clodronate (right). $EGFP^{+}$ neutrophils are shown in green and $tdTomato^{+}$ macrophages are depicted in red. Scale bar, 500µm. Numbers of neutrophils (B) and macrophages (C) in the CHT of 4dpf *mpeg1:tdTomato* larvae 40 hours after liposome injection.

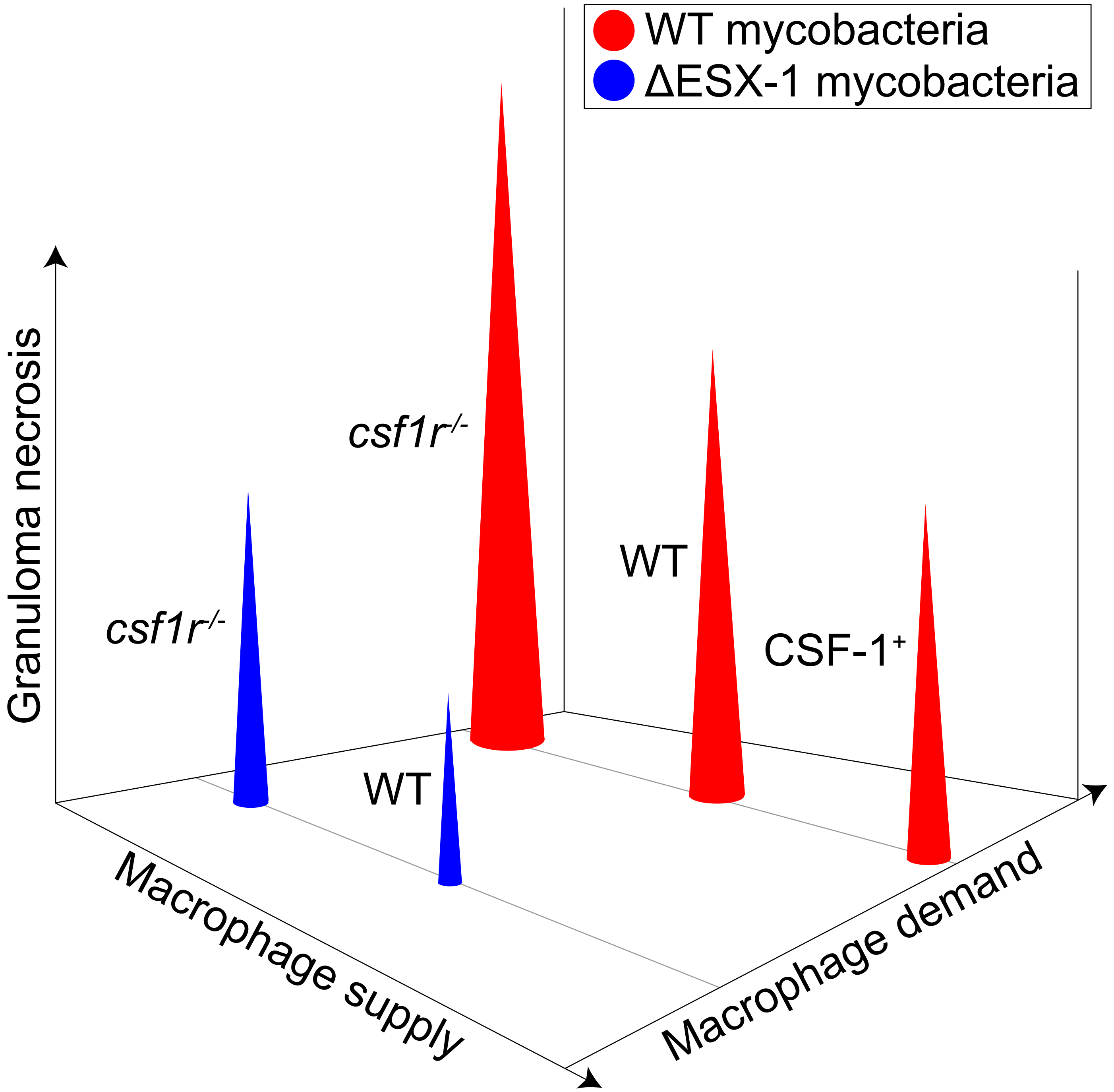

Figure S5. (Related to Figures 2 - 4 and Figure 6). Macrophage demand and supply interact to modulate granuloma necrosis. The relative effects on granuloma necrosis of concurrent variations in macrophage supply and demand tested in this study are shown in a cartoon graph.
